# A microenvironment-responsive FePt probes for imaging-guided Fenton-enhanced radiotherapy of hepatocellular carcinoma

**DOI:** 10.1186/s12951-022-01305-z

**Published:** 2022-03-03

**Authors:** Xingyang Zhao, Xiang Sun, Wenchao Huang, Ronghe Chen, Kang Chen, Liming Nie, Chihua Fang

**Affiliations:** 1 Department of Hepatobiliary Surgery, Zhujiang Hospital, Southern Medical University, Guangzhou, 510280 China; 2 Medical Research Center, Guangdong Provincial People’s Hospital, Guangdong Academy of Medical Sciences, Guangzhou, 510080 China; 3 State Key Laboratory of Molecular Vaccinology and Molecular Diagnosis & Center for Molecular Imaging and Translational Medicine, School of Public Health, Xiamen University, Xiamen, 361102 China; 4 Guangdong Provincial Clinical and Engineering Center of Digital Medicine, Guangzhou, 510280 China

**Keywords:** Molecular imaging, Hepatocellular carcinoma, Fenton-enhanced radiotherapy, MRI switching

## Abstract

Hepatocellular carcinoma (HCC) continues to be one of the most fatal malignancies with increasing morbidity, and potent therapeutics are urgently required given its insensitivity to traditional treatments. Here, we have developed a microenvironment-responsive FePt probes for the highly efficient Fenton-enhanced radiotherapy (FERT) of HCC. The selective release of Fe^2+^ in the acidic tumor microenvironment, but not in normal tissue, together with enhanced levels of hydrogen peroxide produced through the Pt radiosensitization effect, facilitates the generation of an enormous amount of hydroxyl radicals through the Fenton reaction, thereby extending the radiotherapeutic cascade and realizing a powerful therapeutic efficacy for HCC. Moreover, the “burst” release of Fe^2+^ contributes to the T2-to-T1 magnetic resonance imaging (MRI) switching effect, which informs the release of Fe^2+^, making imaging-guided cancer therapy feasible. This work not only breaks the bottleneck of traditional radiotherapy for HCC while minimally affecting normal tissues, but also provides a new strategy for FERT imaging guidance.

**Graphical Abstract:**

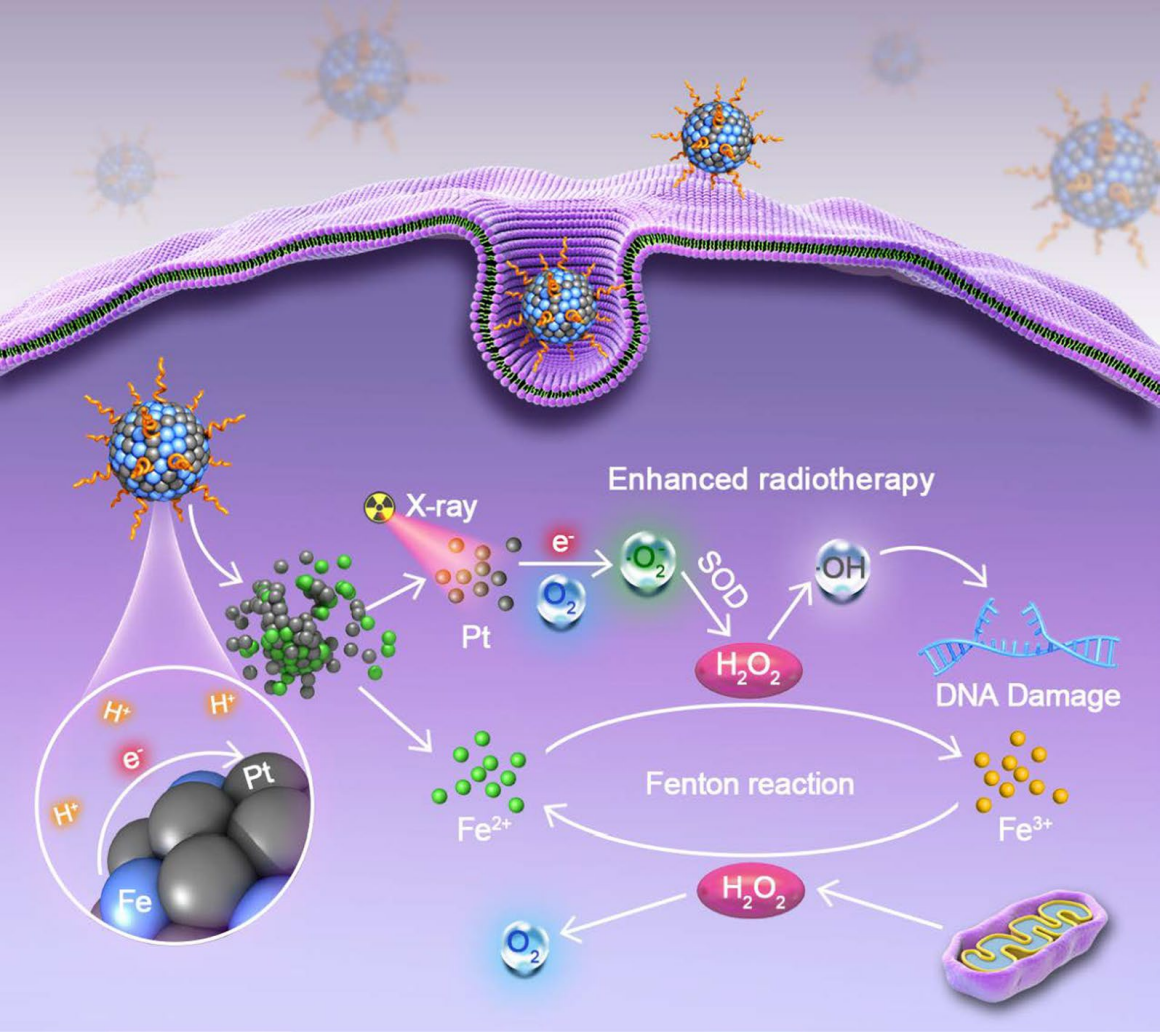

**Supplementary Information:**

The online version contains supplementary material available at 10.1186/s12951-022-01305-z.

## Introduction

Hepatocellular carcinoma (HCC) accounts for the majority (~ 90%) of liver cancers [1], with overall and advanced-stage five-year survival rates of only 18% and 6.5%, respectively [2, 3]. Curative treatment, such as surgical intervention and local ablation, remains the mainstay for early stage HCCs [4–7]. Unfortunately, most patients in the clinic are diagnosed at advanced stages when curative therapies are no longer suitable or effective. In addition, as a remedial treatment for advanced HCC, systemic therapy suffers from obstacles including chemoresistance, severe adverse side effects, as well as drug insufficiency and wastage attributed to metabolism [8–10].

Radiotherapy (RT) has been well-recognized as a competent method for the treatment of cancers for over a century because of its unique advantages [11]. RT can trigger radiological responses in HCC across a wide range of sizes and stages within the liver and palliate extrahepatic metastases [12], being considered an indispensable option for HCC management by the National Comprehensive Cancer Network (NCCN) and the American Association for the Study of Liver Diseases (AASLD) [13, 14]. In addition, for patients with lesions beyond the Milan criteria or for those awaiting liver sources, RT plays important roles in downstaging and bridging HCCs for liver transplantation [15, 16]. However, since radioresistance commonly occurs in HCC, the efficacy of current clinical RT is largely compromised, which results in the inevitable survival of radioresistant HCC cells and, consequently, the recurrence and metastasis of the cancer [17]. Therefore, new strategies that enhance the effectiveness of RT for the treatment of HCC are urgently required.

Radiosensitization with high-Z nanomaterials has achieved milestones in cancer therapy since these materials inherently absorb radiation more than tumor tissue and concentrate the ionization energy in the tumors [18, 19]. However, either through recombination reactions or by the action of superoxide dismutase (SOD), the massively produced reactive oxygen species (ROS) that are mainly responsible for the antitumor effect of RT are eventually transformed into superoxide anion (·O^−^) and hydrogen peroxide (H_2_O_2_) [20, 21], neither of which has been verified as being particularly toxic in vivo since they are rapidly scavenged by over-expressed oxidoreductases in cancer cells [22–24].

Fe^2+^ is an important factor that influences the clinical outcomes of cancer treatment. Low Fe^2+^ levels are associated with poor prognoses in multiple cancers and radioresistance in HCC [25]. Intracellular Fe^2+^ can catalyze the Fenton reaction, which converts H_2_O_2_ into the highly toxic hydroxyl radical (·OH) [26], an aggressive free radical that structurally damages biomolecules, further terminating proliferation and even inducing cancer cell apoptosis [27–29]. Given sufficient H_2_O_2_ and Fe^2+^, adequate ·OH can be generated to impart a curative effect on HCC, making the Fenton reaction an appropriate companion to RT. The formation of ·OH via the Fenton reaction is greatly promoted using the enhanced levels of H_2_O_2_ produced by RT, resulting in powerful cytotoxicity and enhanced RT efficacy.

**Scheme 1:**
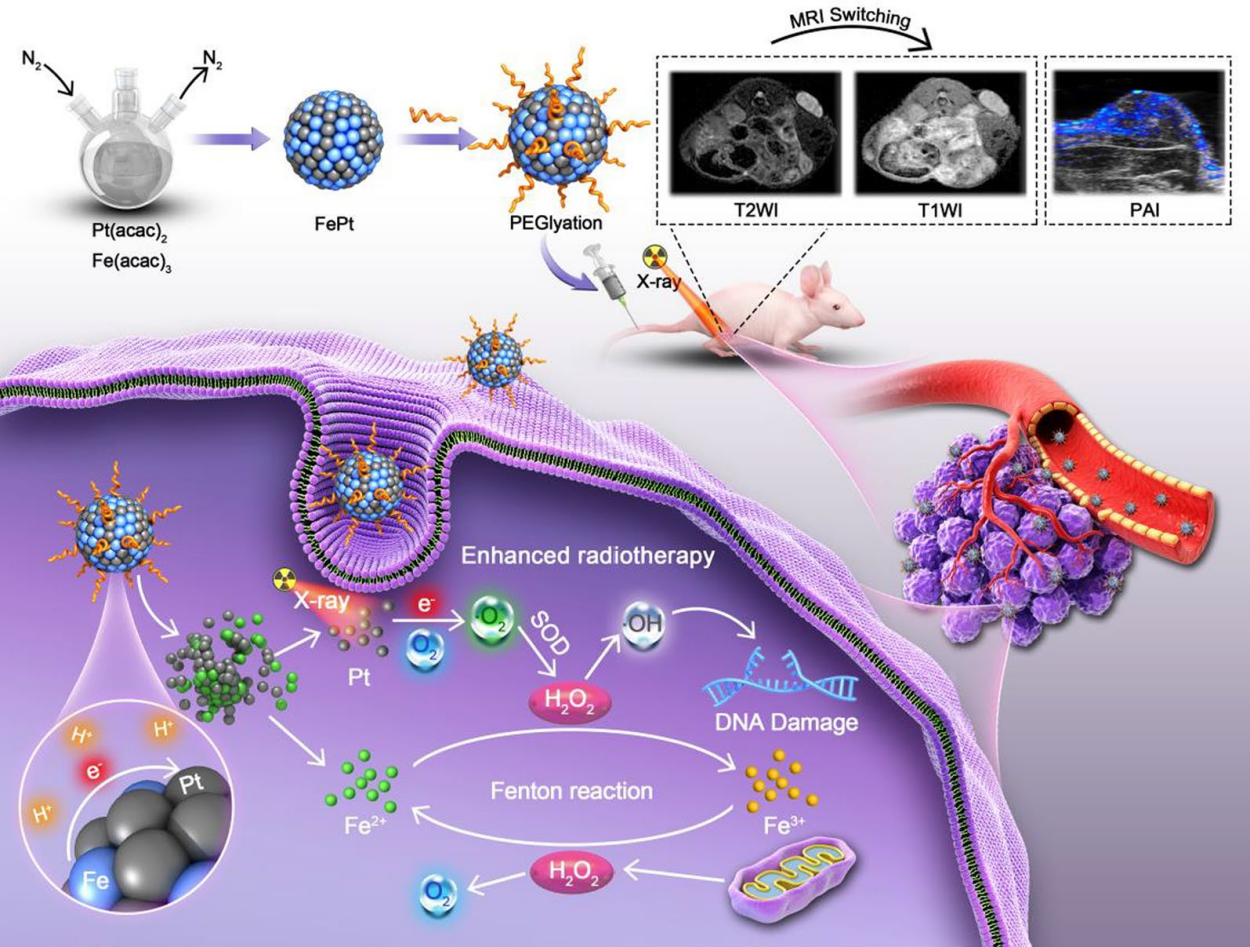
Schematic illustration of the synthesis of FePt nanoprobes, MRI switching and PAI, and the mechanisms of FePt-mediated FERT of HCC

In this study, we synthesized microenvironment-responsive FePt probes for imaging-guided Fenton-enhanced radiotherapy (FERT) of HCC, which promotes the selective generation of ·OH at tumor sites. On the one hand, Pt acts as the radiosensitizer that enhances the yield of H_2_O_2_ in tumor when irradiated with X-rays [30–32], while on the other hand, the “burst” released Fe^2+^ is an excellent catalyst for the Fenton reaction that boosts radiotherapeutic efficacy (Scheme 1). The two initiators, the acidic tumor microenvironment and local X-ray irradiation, which are confined to the tumor tissue, ensure that the therapy is activated only at the tumor site and not in normal tissue, thereby minimizing therapeutic cytotoxicity in non-tumor areas.

In addition to their contrast-enhancing capacities in photoacoustic imaging (PAI), FePt probes also exhibit a magnetic resonance imaging (MRI) switching function, with FePt nanoprobes negatively enhancing T2-weighted imaging (T2WI). As Fe^2+^ release in the acidic tumor microenvironment, the negatively enhanced T2WI is transformed into positively enhanced T1-weighted imaging (T1WI) due to the T1 signal-raising effect of Fe^2+^, which clearly informs the release of Fe^2+^ and, consequently, indicates the generation of ·OH that enables imaging-traceable HCC therapy [33, 34].

We detected the MRI switching effect of FePt nanoprobes using a 9.4 T MRI system, which can serve as the Fe^2+^-release signal in tumors. In addition, we investigated the roles of FePt probes in imaging-guided FERT and determined the mechanism underlying the cascade-extending effect of the Fenton reaction, which enhances radiotherapeutic efficacy.

## Results and discussions

FePt nanoprobes were successfully constructed using a thermo-reduction procedure. Thereafter, polyethylene glycol thiol (mPEG2000-SH) was used as the coating molecule to enhance water stability and biocompatibility. The modified FePt nanoprobes showed remarkably enhanced dispersibility and stability in deionized (DI) water (Additional file 1: Fig. S1). Following this, the intrinsic properties of FePt nanoprobes were investigated.

**Fig. 1:**
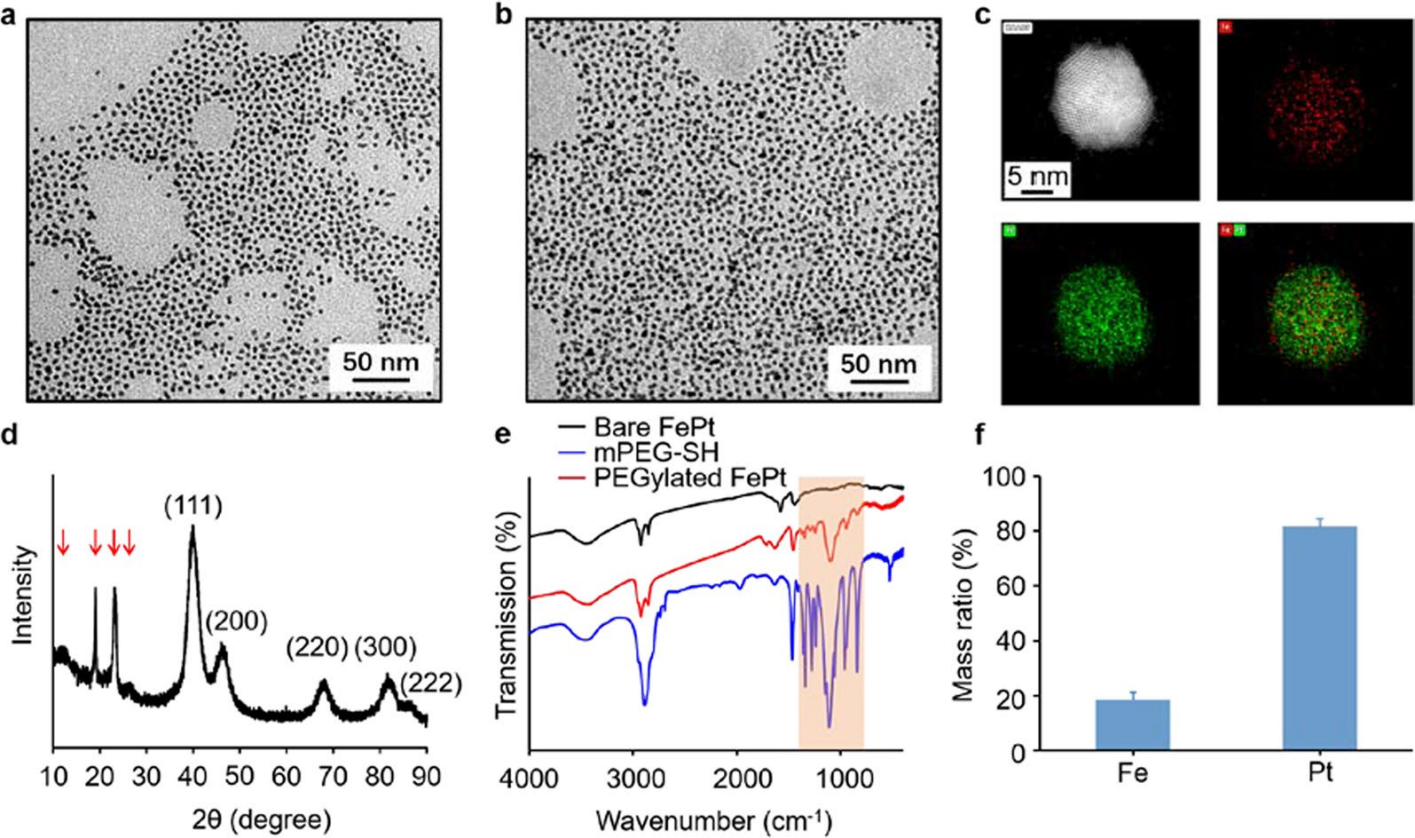
Characterization of the synthesized FePt nanoprobes. **a**, **b** TEM images of FePt nanoprobes before (**a**) and after (**b**) PEGlyation. **c** Elements mapping of FePt nanoprobes (upper left: HAADF, upper right: Pt, lower left: Pt, lower right: merge). **d** XRD pattern of FePt nanoprobes. Red arrow-marked peaks belong to PEG. **e** The FTIR spectra of the bare FePt nanoprobes (black), mPEG-SH (red), and PEGylated FePt nanoprobes (blue). **f** ICP-OES data of FePt nanoprobes quantifying metal ratios

Representative transmission electron microscopy (TEM) images revealed FePt nanoprobes have uniform size distributions (Fig. 1a), and no absolute morphological changes were observed after modification with mPEG2000-SH (Fig. 1b). A representative selection of High-resolution TEM (HRTEM) image showed the morphology of FePt nanoprobes with a diameter of ~ 3.2 nm and the d-spacings for the lattice fringes were ~ 0.22 nm (Additional file 1: Fig. S2). Elements mapping of single and multiple FePt particles indicated Fe and Pt species existed in the nanoparticles (Fig. 1c, Additional file 1: Fig. S3). Meanwhile, the powder X-ray diffraction (XRD) pattern revealed that all diffraction peaks were indexed to FePt with 4 peaks around 20 degrees (red arrows) attributed to PEG were observed (Fig. 1d). Fourier-transform infrared (FTIR) spectroscopy verified that the surfaces of the FePt nanoprobes had been successfully coated with mPEG2000-SH, with specific peaks corresponding to mPEG2000-SH appearing in the spectrum of PEGylated FePt nanoprobes but not in that of the bare FePt nanoprobes (Fig. 1e), where the peak at 1114 cm-1 corresponded to the C–O–C stretching vibration of PEG [35].

The levels of elemental Fe and Pt were quantified by inductively coupled plasma optical emission spectrometry (ICP-OES), which revealed that Fe and Pt accounted for 18.4 wt% and 81.6 wt% of the metal mass, respectively (Fig. 1f). Energy-dispersive X-ray (EDX) spectroscopy was used to further confirm the elemental proportions, with the Fe and Pt ratios determined to be 17.2 wt% and 82.8 wt%, which corresponded to 42.1 at% and 57.9 at%, respectively (Additional file 1: Fig. S4), and is similar to the ICP-OES results. An aqueous dispersion of FePt nanoprobes exhibited a broad-band absorbance spectrum, with no specific absorption peak observed (Additional file 1: Fig. S5a).

Dynamic light scattering (DLS) results showed that FePt nanoprobes have a hydrodynamic size of ~ 7.7 nm (Additional file 1: Fig. S5b). The stabilities of FePt nanoprobes in various media were then evaluated by measuring absorbance spectra between 400 and 900 nm. FePt nanoprobes showed excellent dispersibility and stability in DI water (Additional file 1: Fig. S6a), phosphate buffered saline (PBS) (Additional file 1: Fig. S6b), Dulbecco’s modified Eagle’s medium (DMEM) (Additional file 1: Fig. S6c), and fetal bovine serum (FBS) (Additional file 1: Fig. S6d) for 15 days. The absorbance attributed to FePt nanoprobes in these dispersions remained consistent, which is indicative of excellent dispersibility and stability in the various media. In addition, photographic images of the directly observed dispersions at various time points showed similar results based on color similarity and the absence of precipitation (Additional file 1: Fig. S6e). Ex vivo imaging property investigation under a Vevo 3100 animal PAI system (FUJIFILM VisualSonics, Japan) and a 9.4 T BioSpec 94/30 animal MRI scanner (Bruker, Germany) showed concentration-dependent signal intensities, which exhibited good linearity as a function of FePt concentration (Additional file 1: Fig. S7).

**Fig. 2:**
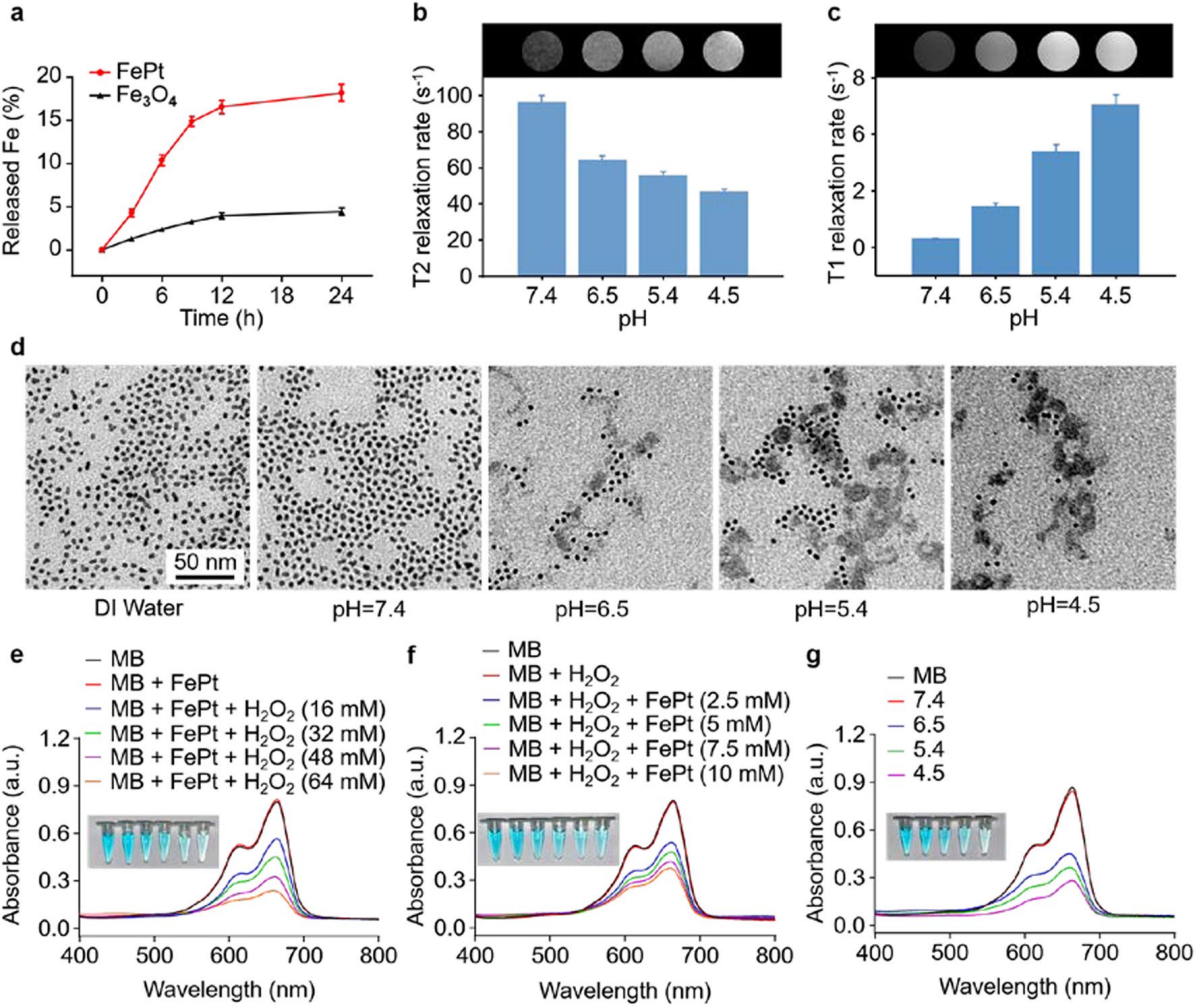
Ex vivo Fe^2+^ release and Fenton catalysis properties of FePt nanoprobes. **a** ICP-OES analyses of Fe^2+^ released from FePt nanoprobes (red) and Fe_3_O_4_ (black). **b** T2 relaxation rates of FePt nanoprobes incubated at various pH. **c** T1 relaxation rates of FePt nanoprobes incubated at various pH. **d** TEM images of FePt nanoprobes under various pH conditions. **e** MB absorbance after incubation with FePt nanoprobes (Fe concentration: 10 mM) and various concentrations of H_2_O_2_ at pH 5.4. **f** MB absorbance after incubation with various concentrations of FePt nanoprobes and H_2_O_2_ (40 mM) at 5.4 pH. **g** MB absorbance after incubation with FePt nanoprobes (Fe concentration: 10 mM) and H_2_O_2_ (40 mM) at various pH

The therapeutic potential of FePt nanoprobes was evaluated in a series of ex vivo tests. First, the amount of Fe released from FePt nanoprobes in an acidic environment was quantified using ICP-OES. FePt nanoprobes released ~ 16.59% Fe between 0 and 12 h, which gradually stabilized at ~ 18.20% at 24 h (Fig. 2a). In comparison, Fe_3_O_4_ NPs, a commonly used Fenton agent, only released ~ 4.43% Fe, which highlights the outstanding Fe-releasing capacity of FePt nanoprobes [36, 37]. In addition, the Fe release of the FePt nanoprobes was compared with that of the 0-valent Fe nanoparticles, indicating a ~ 17.02% versus ~ 11.47% Fe release of FePt and Fe nanoparticles (Additional file 1: Fig. S8). For the reason of the enhanced Fe release of FePt nanoprobes, we hypothesize the phenomenon is attributed to the electron interaction and transfer between Fe and Pt, which results in a different corrsion procedure compared with elemental Fe. In addition, the Fe release of the FePt nanoprobes was facilitated by acidity, in which Fe release increased as pH decreased. Under the pH at 4.5, 5.4, 6.5 and 7.4, the FePt nanoprobes released ~ 18.00%, ~ 12.13%, ~ 8.28% and ~ 1.17% Fe, respectively (Additional file 1: Fig. S9). Meanwhile, no obvious Pt release was detected (Additional file 1: Fig. S10).

In terms of the MRI switching effect, the paramagnetic Fe atoms that remain in the undissociated FePt probes are excellent negative T2WI contrast agents, while the released Fe^2+^ is a positive T1WI enhancer. The Fe^2+^ concentration increased during acid dissociation, while the atomic Fe content decreased simultaneously. According to the MRI data, the T2-weighted relaxation rate in a mild alkaline environment (pH = 7.4) was ~ 96.39 s^−1^, highlighting the potential of these FePt nanoprobes as contrast agents for T2-weighted MRI. Interestingly, lower T2-weighted relaxation rates were observed when FePt nanoprobes were exposed to acidic conditions, with values of ~ 64.11, ~ 55.79, and ~ 46.85 s^−1^ determined at pH 6.5, 5.4, and 4.5, respectively (Fig. 2b). Meanwhile, the T1-weighted relaxation rates of the same mixture increased from ~ 0.31 s^−1^ (pH 7.4) to 1.46 s^−1^ (pH 6.5), 3.38 s^−1^ (pH 5.4), and ~ 5.04 s^−1^ (pH 4.5) (Fig. 2c). These observations indicate that the release of Fe^2+^ can be traced by the T2-to-T1 MRI transition, which provides a potential method for detecting Fe^2+^ release in tumor areas. According to the TEM images, the FePt morphology changed notably to flake-like shadows under acidic conditions, indicating the disintegration of the original structure, while the morphology under alkaline conditions remained similar to that observed in DI water, which provided further evidence that these FePt probes dissociated in acidic conditions (Fig. 2d). In the Fenton reaction, H_2_O_2_ is converted into highly toxic ·OH catalyzed by Fe^2+^. The generated ·OH can efficiently disrupt the structures of biomolecules, such as proteins, lipoids, and DNA, in cancer cells [38]. Methylene blue (MB), a dye that can be decolored by ·OH [39, 40], was used to indicate the generation of ·OH. Significant decreases in MB absorbance were observed when the dye was incubated with FePt probes and H_2_O_2_ under acidic conditions, whereas only slight variations were detected in the absence of FePt nanoprobes, H_2_O_2_, or the low-pH environment (Fig. 2e–g). Electron paramagnetic resonance (EPR) further confirmed the generation of ·OH with a strong four-line signal with a 1:2:2:1 peak-to-peak intensity pattern detected in FePt group, which was similar with the FeCl_2_ group (positive control) but absent in the H_2_O group (negative control) (Additional file 1: Fig. S11). These results demonstrated that the generation of ·OH was accelerated by Fe^2+^ released from FePt nanoprobes in acidic environments when H_2_O_2_ was added.

Prior to FERT application to cells, we assessed the in vitro biosafety of FePt nanoprobes by using 4-nitrophenyl chloroformate 3-(4,5-dimethylthiazol-2-yl)-2,5-diphenyltetrazolium bromide (MTT) assays (Additional file 1: Fig. S12). After incubation with FePt nanoprobes at gradient concentrations for 24 h, L02 cells maintained superior survival rates even at a Fe concentration as high as 3.9 mM, confirming the excellent biocompatibility of FePt nanoprobes, whereas HepG2 cells were somewhat less viable than L02 cells, which is attributable to the higher levels of H_2_O_2_ in cancer cells [41].

**Fig. 3:**
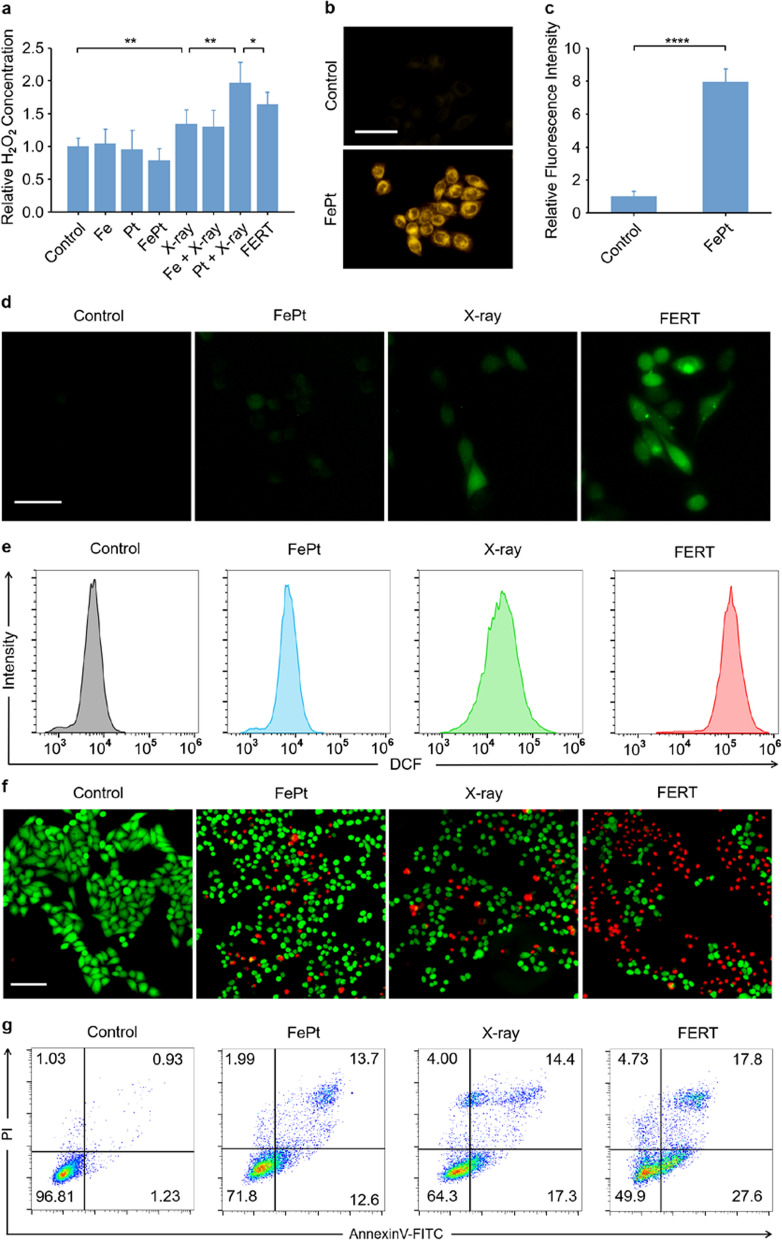
In vitro FERT efficiencies of FePt nanoprobes. **a** Intracellular H_2_O_2_ levels after the indicated treatments. (*P < 0.05, **P < 0.01). **b** Fe^2+^ fluorescence staining without and with FePt nanoprobes treatment (scale bar: 100 μm). **c** Quantification of Fe^2+^ fluorescence intensities. (****P < 0.0001). **d** Intracellular ROS generation detected by the DCFH-DA probe after various treatments. **e** Quantifying ROS using flow cytometry with DCFH-DA detection. **f** Calcein-AM/PI co-staining of HepG2 cells after the indicated treatments (green: live cells; red: dead cells). **g** Flow cytometry analyses of cell apoptosis by Annexin V-FITC/PI co-staining. (Fe concentration: 2.6 mM, X-ray: 4 Gy). FePt nanoprobes and X-ray doses remained consistent throughout this part of the experiment (scale bar: 100 μm)

Then, the experimental treatments to HepG2 cells were performed to further evaluate the therapeutic efficacy of FERT. First, intracellular H_2_O_2_ concentrations were quantified after various treatments to HepG2 cells. Cells exposed to Pt NDs plus X-ray radiation showed the most significant increase in H_2_O_2_ content (Fig. 3a). Nevertheless, the H_2_O_2_ content in the FERT group (FePt nanoprobes plus X-ray irradiation) was significantly lower, which indicates that H_2_O_2_ was decomposed by Fe^2+^ released from FePt probes. Furthermore, the promotion of endocellular Fe^2+^ by FePt nanoprobes was verified using the FerroOrange fluorescence staining method, in which fluorescence intensity was enhanced as the Fe^2+^ was enriched in the cells. Cells treated with FePt nanoprobes exhibited enhanced fluorescence than the control group (Fig. 3b), and the fluorescence quantification of cells treated with FePt nanoprobes increased by ~ 7.96 folds compared with that of control group, revealing that co-incubation with FePt nanoprobes increased the Fe^2+^ concentration in HepG2 cells.

As FePt corrosion leads to Fe^2+^ release, the ROS levels in cells treated under various conditions were then studied using 2′,7′-dichlorodihydrofluorescein diacetate (DCFH-DA), a probe that can be decomposed by ROS into 2′,7′-dichlorofluorescein (DCF), a green fluorescence-emitting molecule [42]. Compared with the control group, the fluorescence intensities of the cells of both the FePt and X-ray groups increased only slightly, while the FERT group exhibited significantly higher fluorescence, which suggests an enhancement in the oxidative stress that was attributed to the higher oxidative activity of ·OH in the latter group. Moreover, flow cytometry, which was used to quantify DCF fluorescence intensity, showed an orders-of-magnitude more intense ROS level in the FERT group than that in other groups, which corroborated the fluorescence microscopy results (Fig. 3e).

Since the excessive production of ROS in cells induces apoptosis, cytotoxicity studies were conducted. The FePt dose-dependent cytotoxicity after X-ray irradiation was illustrated using an MTT assay, which showed a significant decrease in the cell viability at the Fe concentration of 1.3 mM with 4 Gy X-ray irradiation (Additional file 1: Fig. S13). The calcein-AM (live cells, green fluorescence) and propidium iodide (PI, dead cells, red fluorescence) staining method was then employed to visualize the antitumor effect. The images captured using fluorescence microscopy demonstrated the excellent therapeutic efficacy of FERT (FePt nanoprobes plus X-ray irradiation) (Fig. 3f). In contrast, neither the NPs nor X-ray treatment alone was able to effectively kill the cancer cells. The cell-killing effect was further examined using flow cytometry, in which the cells that received various treatment were stained with Annexin V-FITC and PI to quantify cell apoptosis. The results showed that the apoptotic rates of the control, FePt, X-ray, and FERT groups were ~ 3.2%, ~ 28.3%, ~ 35.4%, and ~ 50.1%, respectively (Fig. 3g), which were similar to that of the MTT assay and calcein-AM/PI staining, and highlighted the excellent therapeutic efficacy of FERT. In addition, the treatment effect was further confirmd by colony formation assays, which showed the number of the clone decreased significantly in the FERT group compared with that in the control (**P < 0.01), FePt (**P < 0.01) and X-ray (*P < 0.05) groups, accounting for 33.10% of the number of the control group (Additional file 1: Fig. S14).

In vivo circulation time and biosafety was evaluated to determine the feasibility of administering FePt nanoprobes to animals for in vivo imaging and therapy. The blood circulation time was evaluated by ICP-OES after intravenous injection of the FePt nanoprobes, which was calculated as dose per gram of blood (%ID/g). The results showed a rapid increase after the administration of the nanoprobes, up to ~ 6.49%ID/g at 0.5 h, and gradually decreased to ~ 0.50%ID/g at 24 h, indicating the metabolism of the nanoprobes (Additional file 1: Fig. S15). Healthy mice were intravenously injected with FePt nanoprobes, and serum samples and the main organs (heart, liver, spleen, lung and kidney) were collected on 1, 3, and 7 d post-injection for biochemical and histological examination. Biochemical parameters, including alanine aminotransferase (ALT), aspartate aminotransferase (AST), alkaline phosphatase (ALP), albumin (ALB), and urea levels were measured. Compared with the control group, no obvious differences were observed in any of these indexes at different timepoints and histology revealed no noticeable signs of inflammation or tissue damage in the pathological sections of the major organs (Additional file 1: Fig. S16 and S17). Moreover, hemolysis was investigated to assess the red blood cell (RBC) biosecurity of FePt nanoprobes by using blood samples collected from healthy mice. RBCs treated with DI water were regarded as the positive control and those treated with PBS (Fe concentration = 0 mM) served the negative control. No obvious hemolysis effect resulting from FePt nanoprobes was observed, indicating that FePt nanoprobes are safe for RBCs circulating in blood vessels (Additional file 1: Fig. S18). The results above indicated that FePt nanoprobes are safe to be applied to in vivo experiments.

**Fig. 4:**
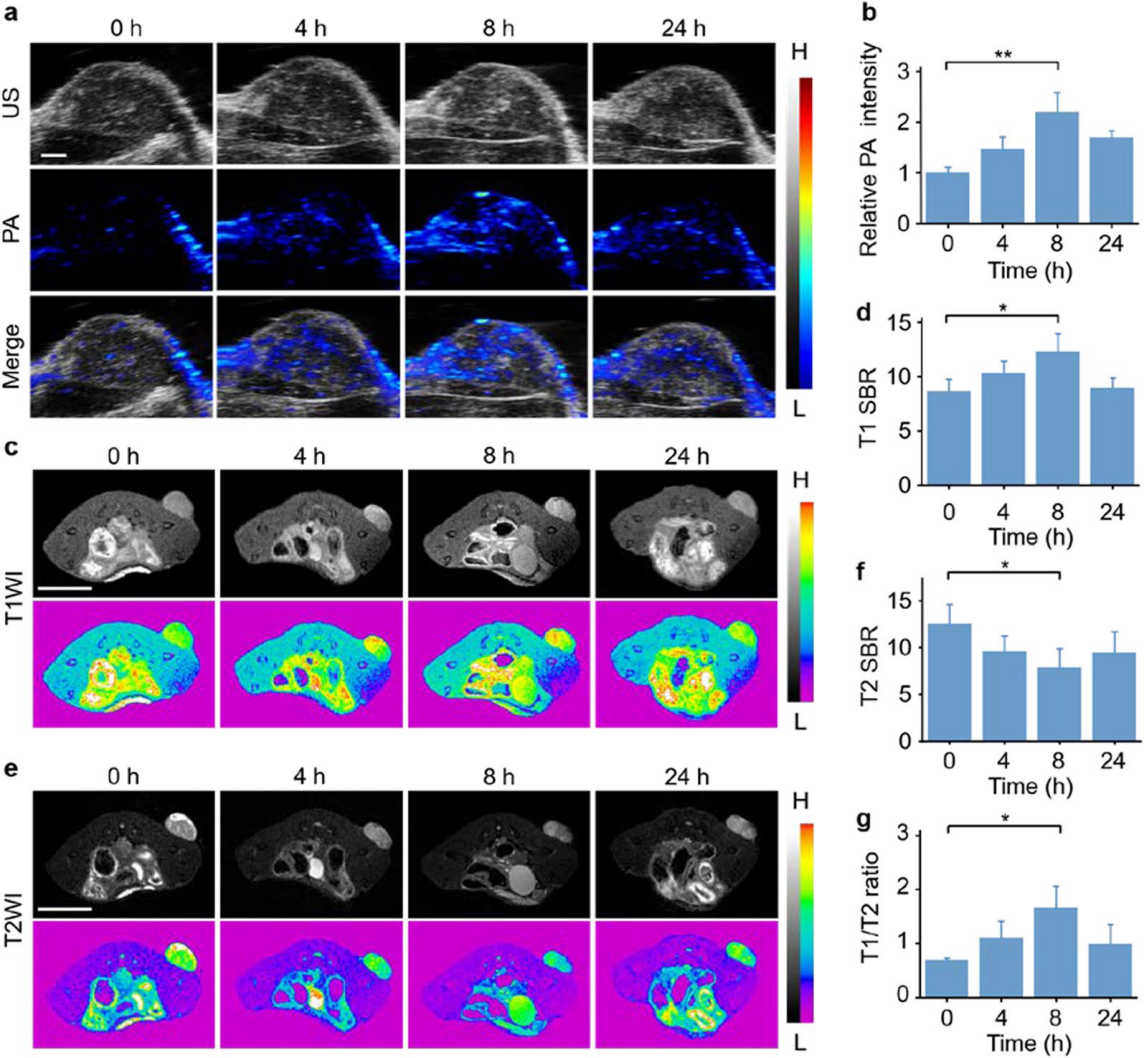
In vivo PAI and MRI of HepG2 tumors. **a**, **b** In vivo PAI of HepG2 tumors at various timepoints post-injection of FePt nanoprobes (scale bar: 1 mm) (**a**), and the corresponding relative photoacoustic intensities in the tumor area (**b**). (**c**, **d**) In vivo T1WI of HepG2 tumors at various timepoints after intravenous injection of FePt nanoprobes (scale bar: 1 cm) (**c**), and the corresponding T1 SBRs of the tumor area (**d**). **e**, **f** In vivo T2WI of HepG2 tumors at various timepoints after intravenous injection of FePt nanoprobes (scale bar: 1 cm) (**e**), and the corresponding T2 SBRs of the tumor areas (**f**). **g**) Ratios of T1 SBR to T2 SBR. (*P < 0.05, **P < 0.01)

As an emerging modality for molecular imaging, PAI exhibited superiority both in morphological and functional imaging [43–45]. MRI is commonly used in clinical setting as a traditional and robust imaging modality. Thus, the performance of FePt nanoprobes as contrast agents for in vivo tumor imaging was evaluated. Based on the excellent performance of FePt nanoprobes in in vitro PAI and MRI, we successfully conducted analogous in vivo imaging. The photoacoustic images showed that the tumor was lightened after injection of FePt nanoprobes (Fig. 4a). The photoacoustic intensities were enhanced by a factor of ~ 2.2 following intravenous administration, demonstrating the potential of FePt nanoprobes as competent photoacoustic contrast agents for tumor imaging (Fig. 4b). According to previous ex vivo MRI switching results, the relaxation rate converted from T2 to T1 during FePt corrosion. Hence, both the T1-weighted and T2-weighted in vivo images of the same sections of tumor-bearing mice were captured, and the signal-to-background ratios (SBRs) at different time points were calculated. The tumors exhibited enhanced T1WI only at tumor sites after intravenous injection of FePt nanoprobes, which indicates Fe^2+^ release at the tumor sites (Fig. 4c, d). Meanwhile, negative enhancement of T2WI was observed in tumor areas (Fig. 4e, f), which is regarded as a sign of FePt nanoprobes retention in these tumors. The ratios of T1 SBR and T2 SBR were calculated, showing a remarkable increase in the ration at 8 h after injection (Fig. 4g). The MRI switching phenomenon detected in vitro was also observed in vivo, thereby offering a valid method for visualizing the release of Fe^2+^ at tumor sites. As a contrast, Fe_3_O_4_ nanoparticles were employed in MRI. Although the SBRs in T2WI sightly decreased due to the accumulation of the Fe_3_O_4_ nanoparticles at the tumor sites, the T1WI SBRs showed no obvious increases (Additional file 1: Fig. S19). The MRI switching phenomenon was not detected when applying the Fe_3_O_4_ nanoparticles, which indicated the outstanding and unique MRI switching ability of the FePt nanoprobes.

**Fig. 5:**
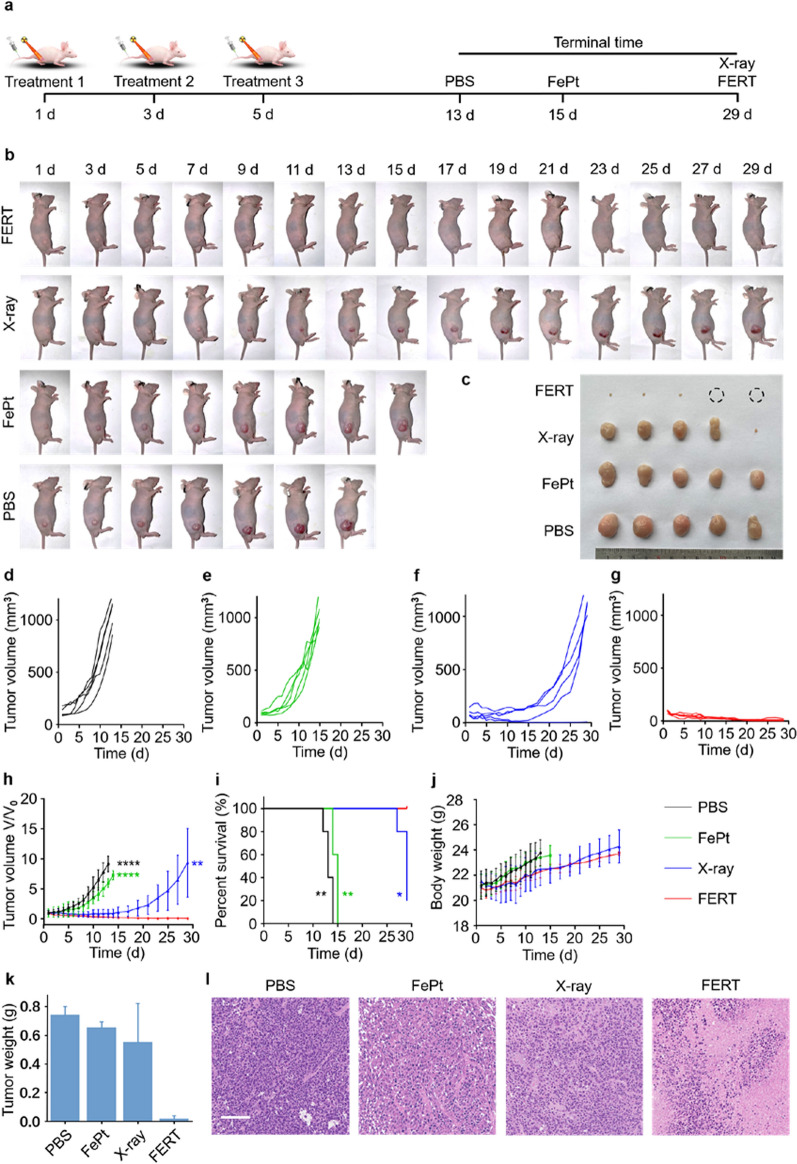
In vivo FERT of HepG2 tumors. **a** Illustration of the treatment procedure. Tumor-bearing mice were treated as indicated. **b** Representative photographic images of mice at various time points. **c** Photographic images of tumors harvested from mice after termination of the monitoring period. **d**–**g** Individual tumor growth curves of the mice in the PBS (**d**), FePt (**e**), X-ray (**f**) groups, and FERT group (**g**). **h** Relative tumor volume curves for the various treatment groups. The statistical differences were derived from the comparison between the corresponding groups (PBS, FePt, X-ray) and the FERT group, respectively. (****P < 0.0001, **P < 0.01). **i** Survival curves of the various groups. The statistical differences were derived from the comparison between the corresponding groups (PBS, FePt, X-ray) and the FERT group, respectively. (**P < 0.01, *P < 0.05). **j** Body weights as functions of time of mice in the various treatment groups. **k** Tumor weights of mice in the various groups. **l** Images of tumor slices stained with H&E collected from various groups of mice (scale bar: 100 μm)

Encouraged by the promising therapeutic performance against HepG2 cells in vitro, the efficacy of FERT for the treatment of tumors in vivo was further evaluated. Tumor-bearing mice were randomly divided into four groups, and received various treatments, including the administration of PBS, FePt nanoprobes, X-ray irradiation, and FePt nanoprobes combined with X-ray irradiation (FERT). Each treatment was performed three times on individuals in the corresponding groups, on days 1, 3, and 5 (Fig. 5a). The monitoring of the PBS and FePt groups was terminated, and the mice were euthanized on days 14 and 15, respectively, at which points the tumors were ~ 1000 mm^3^ in size (Fig. 5b). As shown in the photographic images of tumor-bearing mice in the four groups acquired during therapy, the tumors of the mice in the FERT group showed significantly reduced tumor volumes, whereas the X-ray group showed slow tumor growth, while the FePt and PBS groups showed rapid tumor growth (Fig. 5b, c). Quantitative tumor size analysis of the FERT group revealed a continuous decrease in size with time, while tumors of the other three groups tended to increase in volume (Fig. 5d–h). According to the survival curves, the survival rate of the mice in the FERT group was significantly higher than in the PBS (**P < 0.01), FePt (**P < 0.01), and X-ray (*P < 0.05) groups (Fig. 5i). No behavioral disorders were observed in the mice of each group. In addition, no significant differences were observed in mice’s body weights among the groups (Fig. 5j). The tumor weight in the FERT group was significantly lower than those in the PBS, FePt, and X-ray groups (Fig. 5k). Tissues derived from the tumors were stained with hematoxylin and eosin (H&E) to clarify any changes in the internal structures of tumors that received various treatment regimens (Fig. 5l). Meanwhile, Tunel immunofluorescence was performed to evaluate the apoptosis of the tumors (Additional file 1: Fig. S20). A significant level of cell nuclear disappearance was observed in the tumors of the FERT group, which correlates with the effective therapeutic outcomes demonstrated in other in vivo treatment experiments and highlights the extraordinary antitumor effectiveness of FERT. Furthermore, to evaluate the safety of the diverse treatment regimens, the main organs of mice in each group were also analyzed using H&E staining, which revealed that the cells of various organs showed no obvious damage, highlighting the excellent safety of each treatment protocol (Additional file 1: Fig. S21).

## Conclusions

To summarize, the fabricated ultrasmall FePt nanoprobes can achieve efficient Fe^2+^ release and Fenton catalytic performance, enabling imaging-guided FERT of HCC. FERT assisted with FePt nanoprobes converts the H_2_O_2_ overproduced by the Pt radiosensitization effect into highly cytotoxic ·OH, through Fenton reaction, leading to a remarkable enhancement of the intracellular oxidative stress, which is more intense than that in traditional RT, further inducing effective HCC cell apoptosis. In addition, FERT is highly selective to tumor in vivo with the guarantee of the two initiators, acidic microenvironment and X-ray irradiation confined to cancer areas, thereby minimizing the toxicity to normal tissues. Interestingly, the Fe^2+^ release in tumors can be traced by the MRI switching effect after intravenous injection of FePt nanoprobes, offering great potential for the guidance of FERT. This work ingeniously overcomes the shortcomings of traditional RT and provides an intelligent, accurate, and effective therapeutic option with imaging guidance for HCC. Althoguh the accelerated Fe^2+^ release is observed, the underpinned mechanism is unclear. We hypothesized the phenomenon is attributed to the electron interaction and transfer between Fe and Pt. However, further studies are required to unveal the exact mechanism, which will promote the extensive application of FePt nanoprobes and FERT in the treatment of malignant diseases.

## Supplementary Information

**Additional file 1:** **Figure S1.** Dispersibility of FePt nanoprobes before and after PEGylation in different solvents. **Figure S2.** HRTEM of FePt nanoprobes. Lattice fringes exhibited in the inserted view is 0.22 nm (Scale bar: 2 nm). **Figure S3.** Elements mapping of multiple FePt particles. **Figure S4.** EDX analysis of FePt nanoprobes. (a) The view of SEM for EDX analysis. (b) EDX spectrum of FePt nanoprobes. **Figure S5.** (a) The absorbance spectrum of FePt nanoprobes. (b) Hydrodynamic size distribution of FePt nanoprobes. **Figure S6.** The absorbance spectrum of FePt nanoprobes in DI water (a), PBS (b), DMEM (c) and FBS (d). (e) photographs of different dispersions of FePt nanoprobes at different time points. **Figure S7.** (a) T2 relaxation rate of FePt nanoprobes at various concentrations. (b) PA intensities of FePt nanoprobes at various concentrations. **Figure S8.** Fe release of FePt nanoprobes and 0-valent Fe nanoparticles quantified by ICP-OES. **Figure S9.** ICP-OES analysis of released Fe incubated under different pH for 24 h. **Figure S10.** ICP-OES analysis of released Fe and Pt. **Figure S11.** EPR spectra of ·OH in FeCl_2_ (positive control), H_2_O group (negative control) and FePt groups. **Figure S12.** Cytotoxicity of FePt nanoprobes after the incubation with L02 and HepG2 cells for 24 h. **Figure S13.** Cell viabilities of HepG2 cells after X-ray irradiation (4 Gy) with different concentrations of FePt nanoprobes (*, *P* < 0.05. ***, *P* < 0.001). **Figure S14.** Colony formation assays after different treatments (**, *P* < 0.01. **, *P* < 0.01. *, *P* < 0.05). **Figure S15.** In vivo blood circulation by quantifying Pt concentration at different time points after intravenous injection of FePt nanoprobes. **Figure S16.** Serum biochemical indexes including ALT (a), AST (b), ALP (c), ALB (d) and urea (e) of the mice intravenously injected with FePt nanoprobes. **Figure S17.** H&E staining images of main organs (heart, liver, spleen, lung and kidney) collected from mice after intravenous administration FePt nanoprobes (scale bar is 100 μm). **Figure S18.** Hemolysis assays of FePt nanoprobes. **Figure S19.** In vivo MRI of the tumors after intravenously injection of Fe_3_O_4_ nanoparticles. (a, b) In vivo T1WI of HepG2 tumors at various timepoints (scale bar: 1 cm) (a), and the corresponding T1 SBRs of the tumor area (b). (c, d) In vivo T2WI of HepG2 tumors at various timepoints (scale bar: 1 cm) (c), and the corresponding T2 SBRs of the tumor area (d). **Figure S20.** Tunel immunofluorescence of tumor slices collected from various groups of mice. (Scale bar: 100 μm. **Figure S21.** H&E staining images of main organs (heart, liver, spleen, lung and kidney) collected from mice after intravenous administration FePt nanoprobes (scale bar is 100 μm).
