# Supplementary material for: A microenvironment-responsive FePt probes for imaging-guided Fenton-enhanced radiotherapy of hepatocellular carcinoma
Source: J Nanobiotechnology. 2022 Mar 3;20:100. doi: 10.1186/s12951-022-01305-z (PMC8892710; doi:10.1186/s12951-022-01305-z)
Supplement: Supplementary file 1 — Additional file 1: Figure S1. Dispersibility of FePt nanoprobes before and after PEGylation in different solvents. Figure S2. HRTEM of FePt nanoprobes. Lattice fringes exhibited in the inserted view is 0.22 nm (Scale bar: 2 nm). Figure S3. Elements mapping of multiple FePt particles. Figure S4. EDX analysis of FePt nanoprobes. (a) The view of SEM for EDX analysis. (b) EDX spectrum of FePt nanoprobes. Figure S5. (a) The absorbance spectrum of FePt nanoprobes. (b) Hydrodynamic size distribution of FePt nanoprobes. Figure S6. The absorbance spectrum of FePt nanoprobes in DI water (a), PBS (b), DMEM (c) and FBS (d). (e) photographs of different dispersions of FePt nanoprobes at different time points. Figure S7. (a) T2 relaxation rate of FePt nanoprobes at various concentrations. (b) PA intensities of FePt nanoprobes at various concentrations. Figure S8. Fe release of FePt nanoprobes and 0-valent Fe nanoparticles quantified by ICP-OES. Figure S9. ICP-OES analysis of released Fe incubated under different pH for 24 h. Figure S10. ICP-OES analysis of released Fe and Pt. Figure S11. EPR spectra of ·OH in FeCl2 (positive control), H2O group (negative control) and FePt groups. Figure S12. Cytotoxicity of FePt nanoprobes after the incubation with L02 and HepG2 cells for 24 h. Figure S13. Cell viabilities of HepG2 cells after X-ray irradiation (4 Gy) with different concentrations of FePt nanoprobes (*, P < 0.05. ***, P < 0.001). Figure S14. Colony formation assays after different treatments (**, P < 0.01. **, P < 0.01. *, P < 0.05). Figure S15. In vivo blood circulation by quantifying Pt concentration at different time points after intravenous injection of FePt nanoprobes. Figure S16. Serum biochemical indexes including ALT (a), AST (b), ALP (c), ALB (d) and urea (e) of the mice intravenously injected with FePt nanoprobes. Figure S17. H&E staining images of main organs (heart, liver, spleen, lung and kidney) collected from mice after intravenous administration FePt nanoprobes (s [file 12951_2022_1305_MOESM1_ESM.docx]

A Microenvironment-responsive FePt Probes for Imaging-guided Fenton-enhanced Radiotherapy of Hepatocellular Carcinoma

Xingyang Zhao, Xiang Sun, Wenchao Huang, Ronghe Chen, Kang Chen, Liming Nie*, and Chihua Fang^*^

**Experimental**

1. **Materials**

Platinum acetylacetone, iron acetylacetone, oleic acid, oleylamine and SH-PEG2000 were purchased from Aladdin. Dioctyl Ether was purchased from J&K scientific. All reagents were analytical grade and used without further purification.

1. **Synthesis of FePt nanoprobes**

FePt nanoprobes were synthesized via a thermo-reduction procedure. Briefly, 290 mg Platinum acetylacetone was mixed with 353 mg Iron acetylacetone, 774 mg 1,2-hexadecandiol, 4 mL oleic acid, 4 mL oleylamine and 4 mL dioctyl ether in a three-necked flask. Afterwards, the mixture was heated to 100 ℃ (heating rate ~15 ℃/min) under the protection of a gentle N_2_ flow. After 20 min, the reaction system was heated to 240 ℃ with a heating rate of 15 ℃/min and maintained for 1 hour before it was cooled to room temperature. The product was washed with chloroform and ethanol for 3 times and stored in chloroform for further modification.

1. **Surface modification of FePt nanoprobes**

50 mg SH-PEG2000 was dissolved in 10 mL chloroform by sonication, following an addition of 10 mg FePt nanoprobes dispersed in 10 mL chloroform. The mixture was kept in a 55 ℃ water bath for 6 h. Thereafter, the product was washed with ethanol and ultrapure water successively to remove the physically adsorbed ligands on the particle surface. After freeze-drying, FePt nanoprobes were stored in N_2_ atmosphere for further use.

1. **Characterization**

Transmission electron microscopy (TEM) and high-resolution TEM (HRTEM) images were acquired on a Talos F200X transmission electron microscope (FEI, USA). Elements mapping was performed using a Themis Z aberration-corrected scanning transmission electron microscope (ac-STEM, FEI, USA). Energy-dispersive X-ray spectroscopy (EDX) spectra were acquired on an EX-250 EDX analyzer (HORIBA, Japan). X-ray diffraction (XRD) mapping was performed on an Ultima IV X-ray diffractometer (Rigaku Corporation, Japan). Dynamic light scattering was measured using a SZ-100 particle size analyzer (HORIBA, Japan). Optical absorbance and stability were recorded by a Multiskan GO 1050 microplate reader (Thermo Scientific, USA). Fourier transform infrared spectra (FTIR) was investigated on a Nicolet Nexus 470 FTIR spectrometer (Thermo Scientific, USA). The mass percentage of Fe and Pt element was quantified by a SPECTROBLUE FMX36 inductively coupled plasma optical emission spectrometer (ICP-OES, SPECTRO, Germany).

1. ***Ex vivo* Fe release and MRI switching experiments**

FePt nanoprobes containing 1 mg Fe were dispersed in 1 mL HNO_3_ solution (pH = 5.4) in dialysis tubing (MWCO = 1 kDa, Spectrum Laboratories, Inc.) and the filtrate was sampled. The Fe and Pt concentrations in the filtrates were quantified with ICP-OES analysis. Fe_3_O_4_ and 0-valent Fe nanoparticles were used as control. Then, the similar procedures were repeated in HNO_3_ solution (pH = 4.5, 5.4, 6.5, 7.4) and the filtrates were collected with 4 h intervals for Fe concentration quantification with ICP-OES.

For the MRI switching experiment, the T1- and T2- relaxation rates of the FePt nanoprobe dispersions in different pH (pH = 7.4, 6.5, 5.4, 4.8) were measured by a 9.4T BioSpec 94/30 animal MRI scanner (Bruker, Germany).

1. **Acid corrosion of FePt nanoprobes**

FePt nanoprobes were fully dispersed in PBS of various pH (7.4, 6.5, 5.6, 4.5) and incubated in 37 ℃ for 24 h. Then, the samples were collected for TEM observation.

1. **·OH generation of FePt nanoprobes**

PBS (pH = 5.4) solutions of MB (10 μg/mL) containing FePt nanoprobes (Fe concentration: 10 mM) and different concentrations of H_2_O_2_ (0, 16, 32, 48, and 64 mM) was incubated in a 37 ℃ aqueous bath for 30 min. Then, the obtained solutions were centrifuged at 6,000 rpm for 20 min in ultrafilters (Millipore, MWCO = 1 kDa). Afterwards, the absorbance changes of MB were measured. To investigate the influence of Fe concentration on the production of **·**OH, the absorbance of MB in PBS (pH = 5.4) treated with gradient Fe concentrations (0, 2.5, 5, 7.5, 10 mM) and H_2_O_2_ (40 mM) was measured. To investigate the influence of pH values on the formation of **·**OH, MB was incubated with FePt nanoprobes and H_2_O_2_ in different pH values (7.4, 6.5, 5.4, 4.5).

For electron paramagnetic resonance (EPR) analysis, the FePt nanoprobes (Fe concentration: 10 mM, 125μL) and the H_2_O_2_ solution (10mM, 0.5 mL, pH = 4.5) were added successively into DMPO (100 mM, 2ml). After incubation for 30 min, the samples were tested under a EPR instrument (Bruker EMXnano, Germany). FeCl_2_ and H_2_O were employed as positive and negative control.

1. ***Ex vivo* PAI/MRI Study**

Dispersions of FePt nanoprobes at gradient concentrations were used for evaluation of *in vitro* imaging performance under different modalities. For PAI, the samples (6.25, 12.5, 25, 50, 100, and 200 μg/mL) were tested using a Vevo 3100 animal PAI system (FUJIFILM VisualSonics, Japan). For MRI, dispersions (Fe concentration: 0, 0.225, 0.45, 0.9, 1.8, and 3.6 mM) were analyzed using a 9.4 T BioSpec 94/30 animal MRI scanner (Bruker, Germany).

1. **Cytotoxicity**

Hepatocellular carcinoma cells (HepG2) and hepatic cells (L02) were cultured in high-glucose Dulbecco’s Modified Eagle Medium (DMEM, Gibco), supplemented with 10% fetal bovine serum (FBS, Gibco) and 1% penicillin/streptomycin (pen/strep, Gibco) in a 37 °C humidified incubator (Thermo scientific, USA) with 5% CO_2_. L02 and HepG2 cells were seeded in 96-well plates (5,000 cells in 100 μL medium per well) and incubated for 12 h to allow their adherence. After treatment with FePt nanoprobes at gradient concentrations, the cells were incubated for another 24 h. The cell viabilities were measured with MTT assay (Bio Basic, Canada). The measurements were based on absorbance at 570 nm.

1. **H_2_O_2_ concentration assays**

Intracellular H_2_O_2_ concentration was evaluated with an H_2_O_2_ concentration assay kit (Solarbio, China). Briefly, HepG2 cells were seeded into 6-well plates (1×10^6^ cells per well). After allowing them to completely attach, cells received different treatments and were divided into the following groups: control, Fe, Pt, FePt, X-ray, Fe + X-ray, Pt + X-ray and FERT. The H_2_O_2_ concentration was determined according to the manufacturer-recommended procedures. The experiment of each group was performed in triplicate.

1. **Intracellular Fe^2+^ fluorescence staining**

HepG2 cells were seeded into 12-well plates (1×10^5^ cells per well) and subjected to co-incubation with FePt nanoprobes (Fe concentration: 1.3 mM) for 6 h. Afterwards, the cells were stained with FerroOrange (Dojindo, Japan) and observed under a fluorescence microscope.

1. **Intracellular ROS induction**

HepG2 cells were seeded into 12-well plates (1×10^5^ cells per well) and then treated with FePt nanoprobes (Fe concentration: 1.3 mM) and/or X-ray irradiation (2 Gy) as different groups: control, FePt, X-ray and FERT. The cells were stained with DCFH-DA (Beyotime, China) for the fluorescence microscope observation. To quantitatively analyze the fluorescence intensity, the cells stained with DCFH-DA were tested using a CytoFLEX flow cytometer (BECKMAN COULTER, USA).

1. **Cell apoptosis**

HepG2 cells were seeded into 96-well plates (5×10^3^ cells per well). After allowing their adherence, the cells were treated with disparate treatments and divided into following groups: control, FePt, X-ray, FERT. Cell viabilities after treatments were evaluated via MTT assay. For microscopy evaluation, HepG2 cells were seeded into 12-well plates (1×10^5^ cells per well). The Annexin V-FITC and PI cell co-staining strategy (Dojindo, Japan) was employed to evaluate apoptotic rates of cells after different treatments. Calcein-AM and propidium iodide (PI) co-staining method (Beyotime, China) was employed to observed cell apoptosis under a fluorescence microscope (Nikon, Japan). All the experiments were operated according to the manufacturer-provided procedure.

1. **Colony formation assay**

The colony formation assay was performed according to the following procedure: the HepG2 cells were seeded into 6-well plates (2000 cells per well) for 12 h to allow the cells adherence. Then, the cells received the treatments of the nanoprobes (Fe concentration: 1.3 mM) for 24 h together with or without X-ray (2 Gy) and then were divided into control, FePt, X-ray and FERT groups. After 10-day incubation, the cells were stained with 0.1% crystal violet solution. The clone number were quantified with ImageJ (1.46r, National Institutes of Health, USA) and calculated into the ratios between clone number of various groups and that of control group.

1. ***In vivo* biosafety analysis**

All animal experiment protocols were approved by the ethics committee of Zhujiang Hospital, and complied with all relevant ethical regulation. Five-week-old male BALB/c nude mice were purchased from Shanghai SLAC Laboratory Animal Co., Ltd. (China). Healthy mice were intravenously injected with FePt nanoprobes (20 mg/kg). Then, the blood samples were collected on the 1, 3, 7 days after the administration, followed by centrifugation at 4 ℃ and 6, 000 rpm for 10 min to obtain the serum. The blood biochemical parameters including alanine aminotransferase (ALT), aspartate aminotransferase (AST), alkaline phosphatase (ALP), albumin (ALB), and urea were tested. The blood samples from the untreated mice served as the control group. Meanwhile, the main organs (Heart, liver, spleen, lung, and kidney) were harvested for H&E staining.

1. **Blood circulation time analysis**

The blood samples of mice (n = 3 for each time point) were collected from hearts at 0.5, 1, 2, 4, 8, 12 and 24 h after the injection of the nanoprobes. After degradation by aqua regia and filtration through 0.22 μm polyether sulfone membranes, the Pt concentration of the samples were quantified by ICP-OES to calculate the percent injected dose per gram of blood (%ID/g).

1. **Hemolysis analysis**

Fresh blood (1.0 mL) was collected into an anticoagulation tube containing ethylene diamine tetraacetic acid (EDTA) with an addition of 2 mL PBS. After centrifugation (3,000 rpm, 10 min, 4 ℃) and washing with PBS for 3 times, the obtained red blood cells (RBCs) were dispersed in 10 mL PBS. Then, the RBC dispersions (0.2 mL) were gently mixed and incubated with 0.8 mL DI water and FePt dispersions at different concentrations (Fe concentrations: 0, 0.325, 0.65, 1.3, 2.6, 3.25, 3.9 mM) at room temperature for 6 h. The samples were centrifuged (3,000 rpm, 10 min), and the supernatants were collected for the absorbance measurements at 577 nm. Eventually, the hemolysis percent of each sample was calculated.

1. ***In vivo* PA/MR imaging**

Tumor bearing mice were intravenously injected with FePt nanoprobes (5 mg/kg) under anesthetization (2% isoflurane) and PAI was performed at different timepoints. MRI was performed after intravenous administration of FePt dispersion (10 mg/kg) for each mouse. All the instruments for *in vivo* imaging were the same facilities used for *in vitro* imaging. Three mice were recruited for each imaging modality and the signal intensities of tumor areas were analyzed.

1. ***In vivo* FERT against HCC**

HepG2 tumor bearing mice were randomly divided into 4 groups (n = 5) and subjected to different treatments: PBS, FePt nanoprobes (20 mg/kg), X-ray irradiation (4 Gy), and FePt nanoprobes followed by X-ray irradiation. The treatment for each group was repeated 3 times on 1, 3, and 5 days. The tumor volumes were calculated according to the following formula:$\text{ tumor volume}\text{ }\text{=}\text{ }\frac{\text{ab}^{\text{2}}}{\text{2}}$, where the “a” represented the length and the “b” represented the width of the tumors. The tumors and main organs (heart, liver, spleen, lung, and kidney) were resected after the monitoring was terminated and stained with H&E. Mice with tumors larger than ~1,000 mm^3^ were euthanized according to the standard animal protocol.


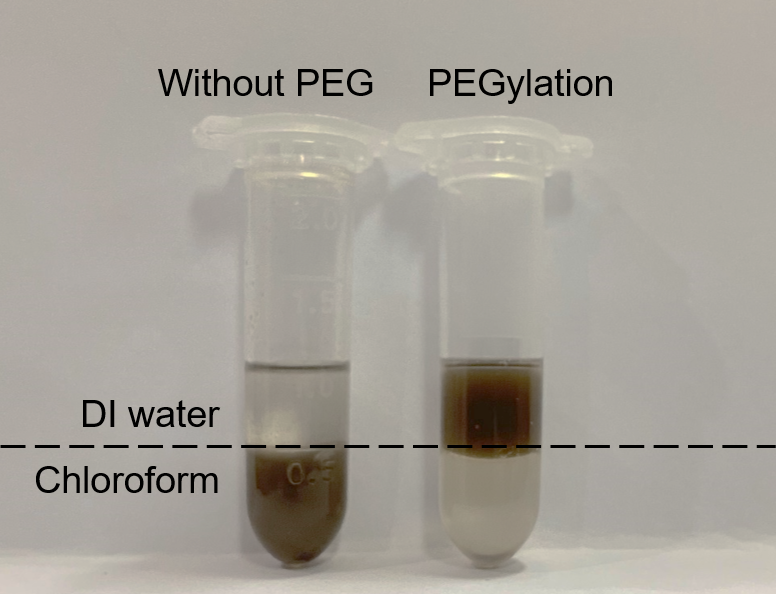


Figure S1. Dispersibility of FePt nanoprobes before and after PEGylation in different solvents.


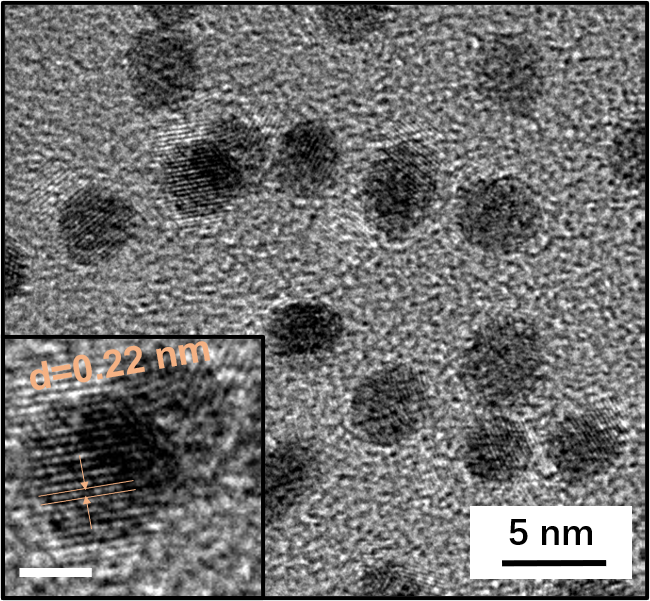


Figure S2. HRTEM of FePt nanoprobes. Lattice fringes exhibited in the inserted view is 0.22 nm (Scale bar: 2 nm)


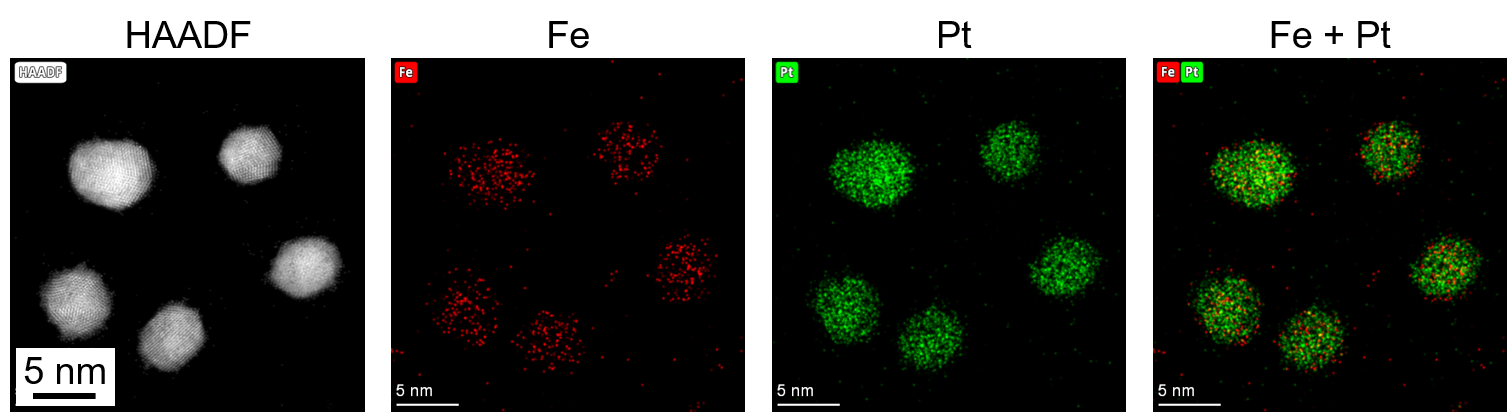


Figure S3. Elements mapping of multiple FePt particles.


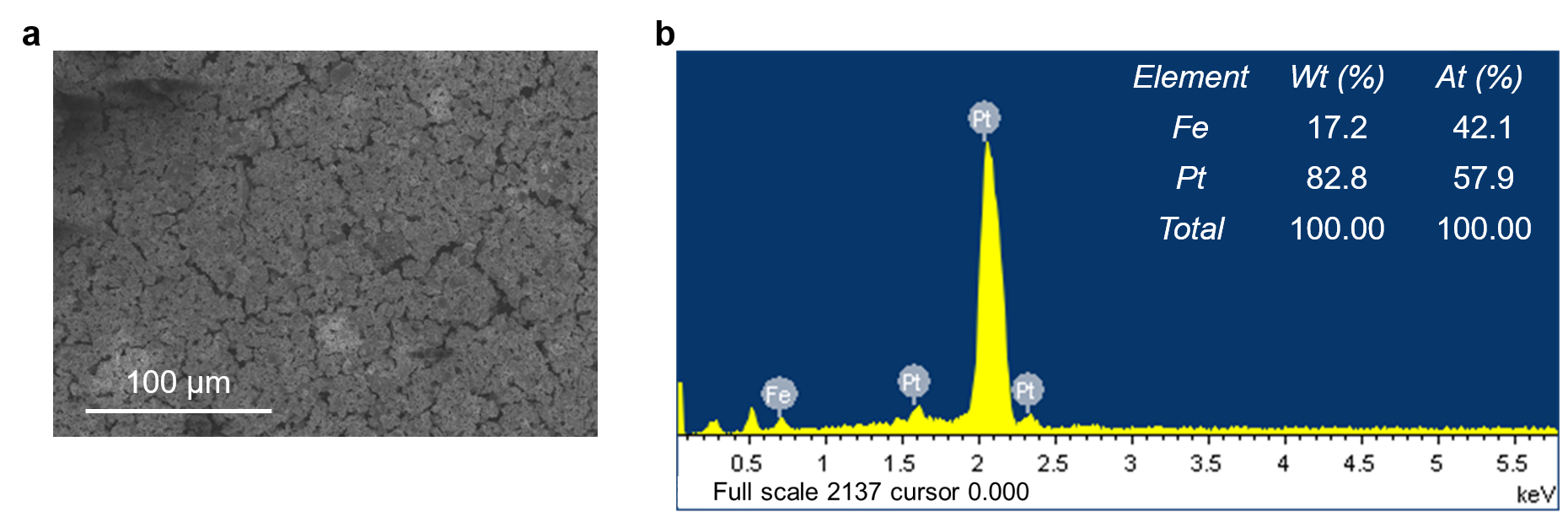


Figure S4. EDX analysis of FePt nanoprobes. (a) The view of SEM for EDX analysis. (b) EDX spectrum of FePt nanoprobes.


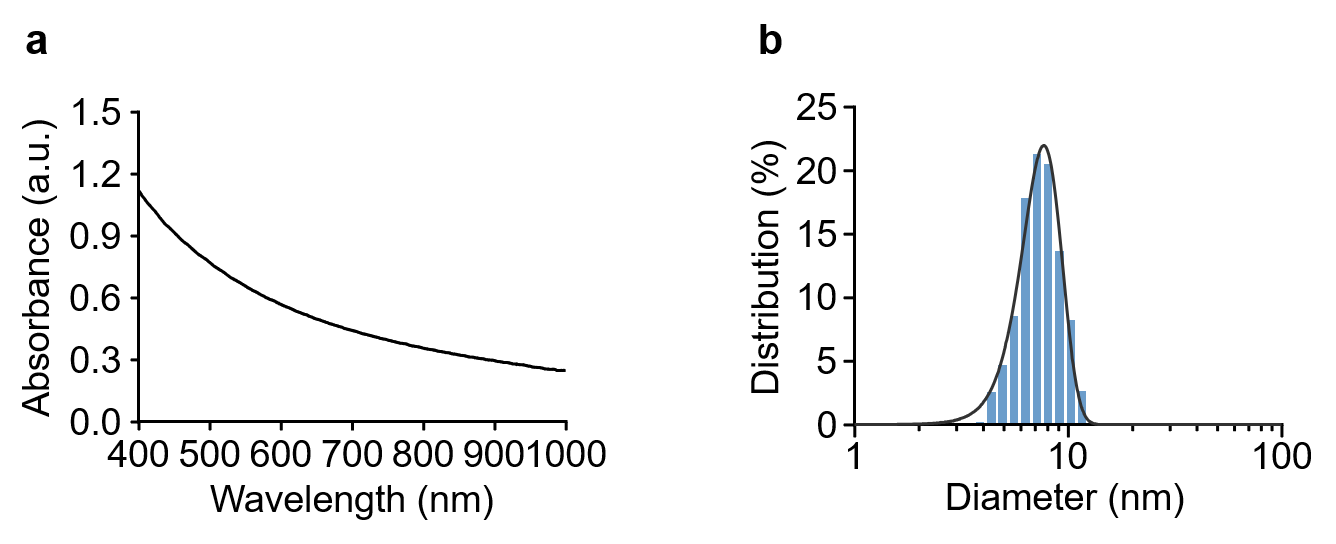


Figure S5. (a) The absorbance spectrum of FePt nanoprobes. (b) Hydrodynamic size distribution of FePt nanoprobes.


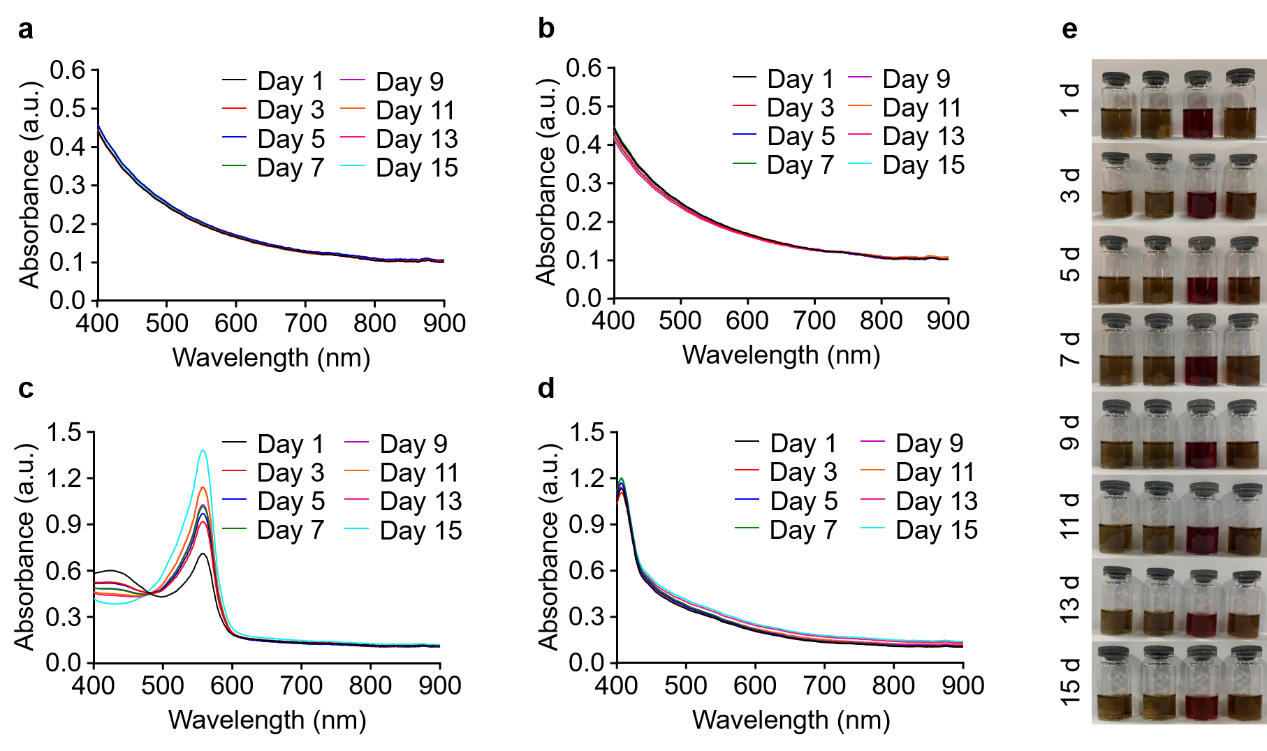


Figure S6. The absorbance spectrum of FePt nanoprobes in DI water (a), PBS (b), DMEM (c) and FBS (d). (e) photographs of different dispersions of FePt nanoprobes at different time points.


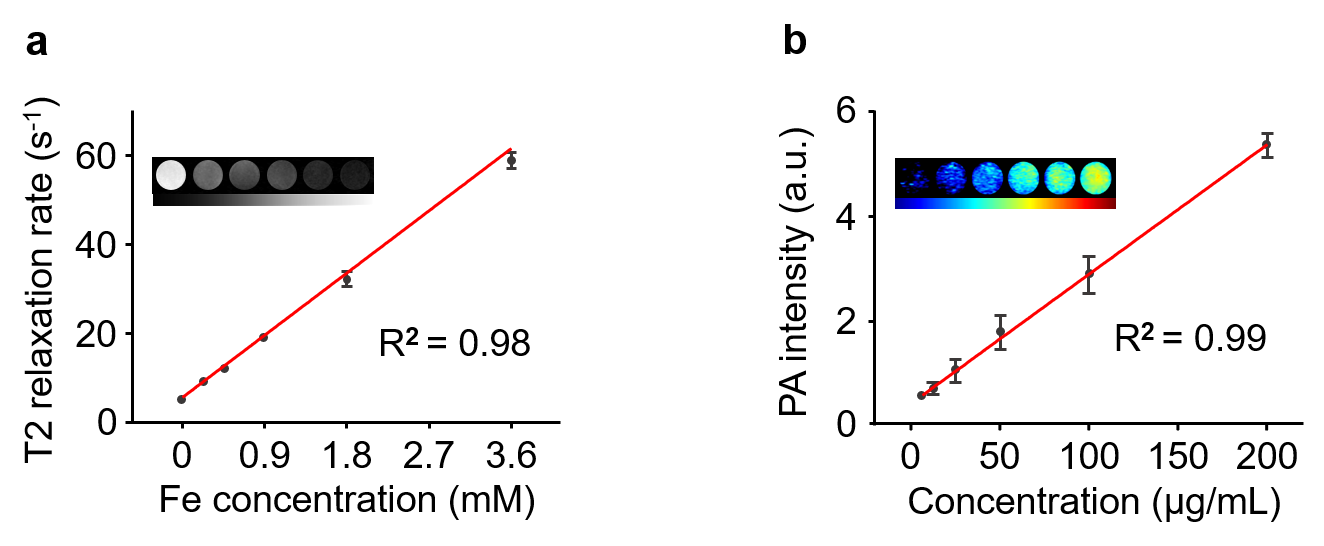


Figure S7. (a) T2 relaxation rate of FePt nanoprobes at various concentrations. (b) PA intensities of FePt nanoprobes at various concentrations.


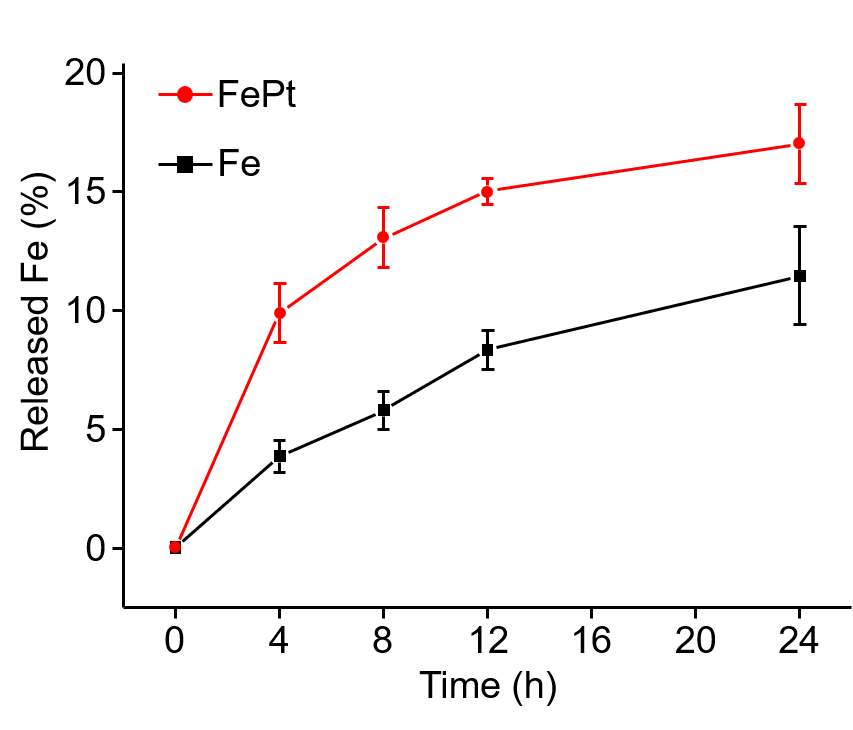


Figure S8. Fe release of FePt nanoprobes and 0-valent Fe nanoparticles quantified by ICP-OES.


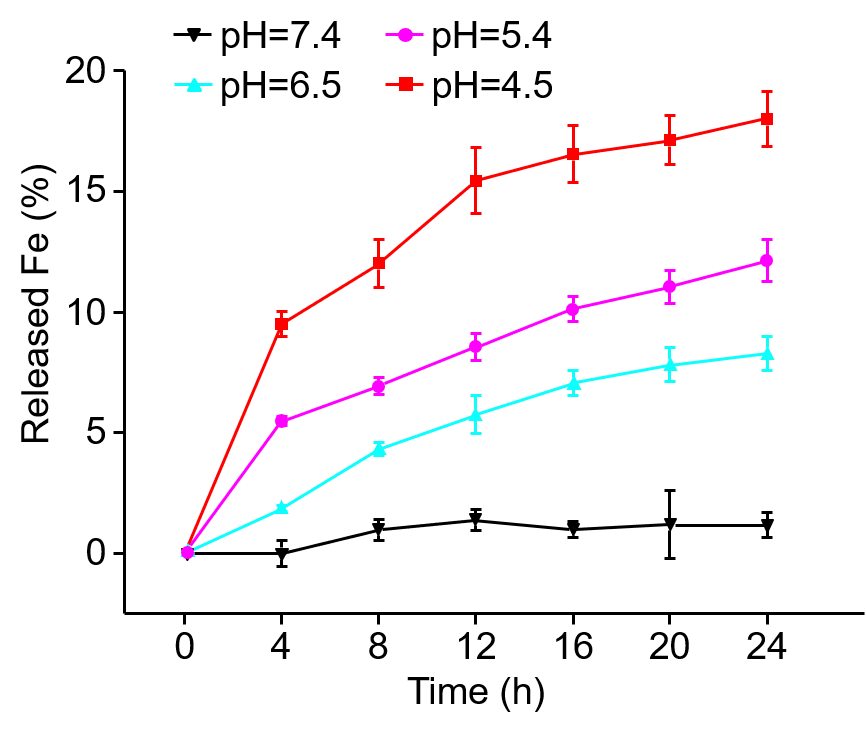


Figure S9. ICP-OES analysis of released Fe incubated under different pH for 24 h.


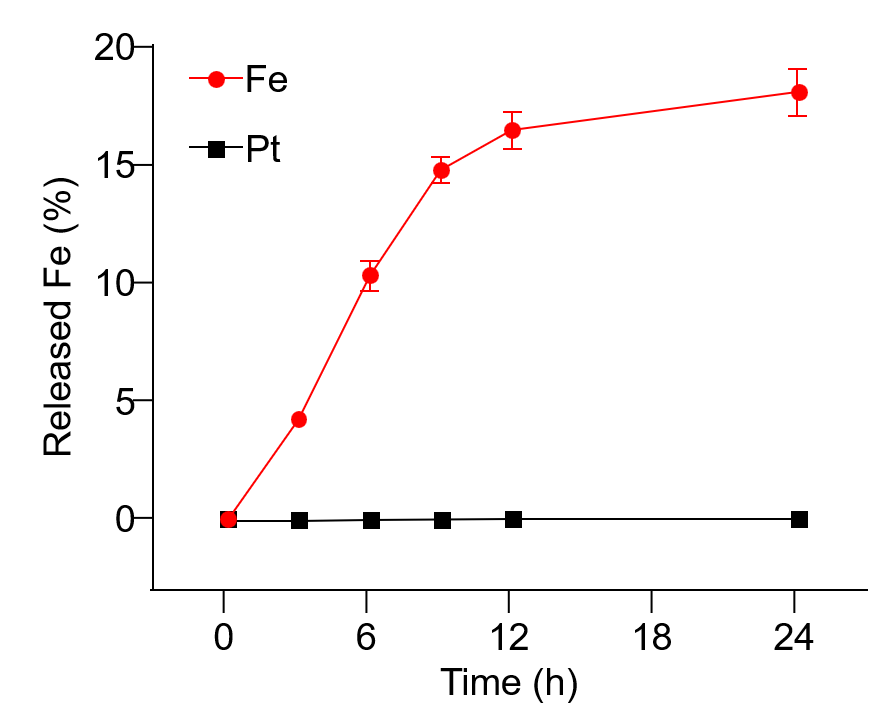


Figure S10. ICP-OES analysis of released Fe and Pt.


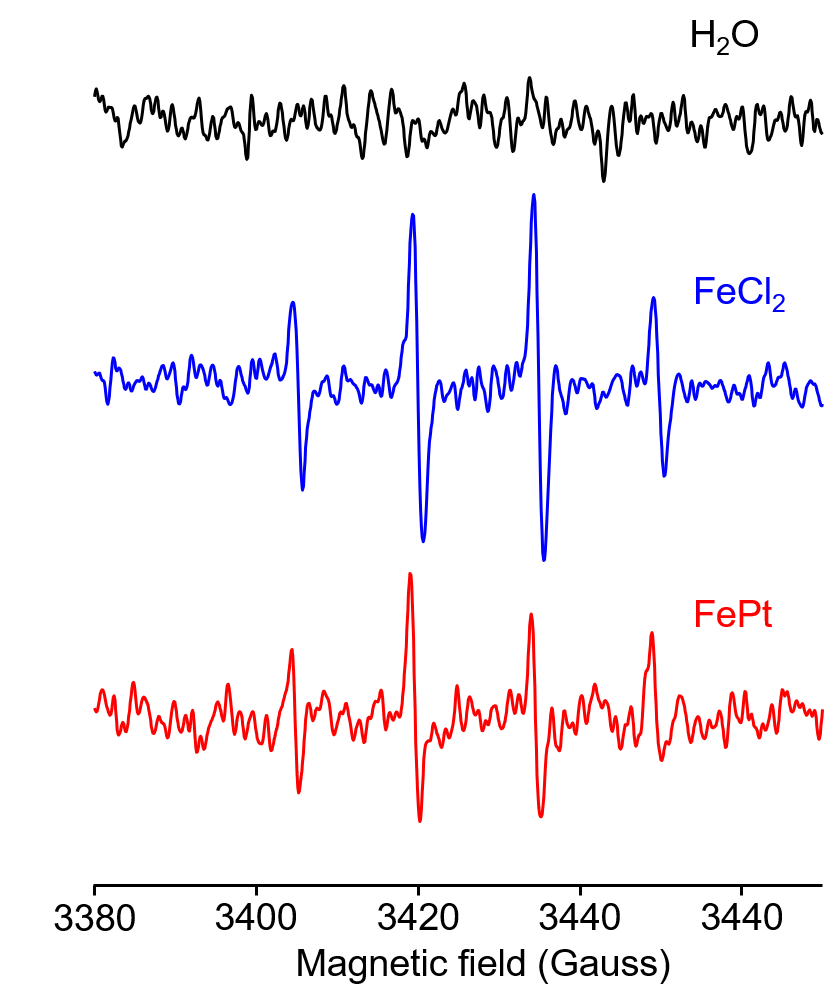


Figure S11. EPR spectra of ·OH in FeCl_2_ (positive control), H_2_O group (negative control) and FePt groups.


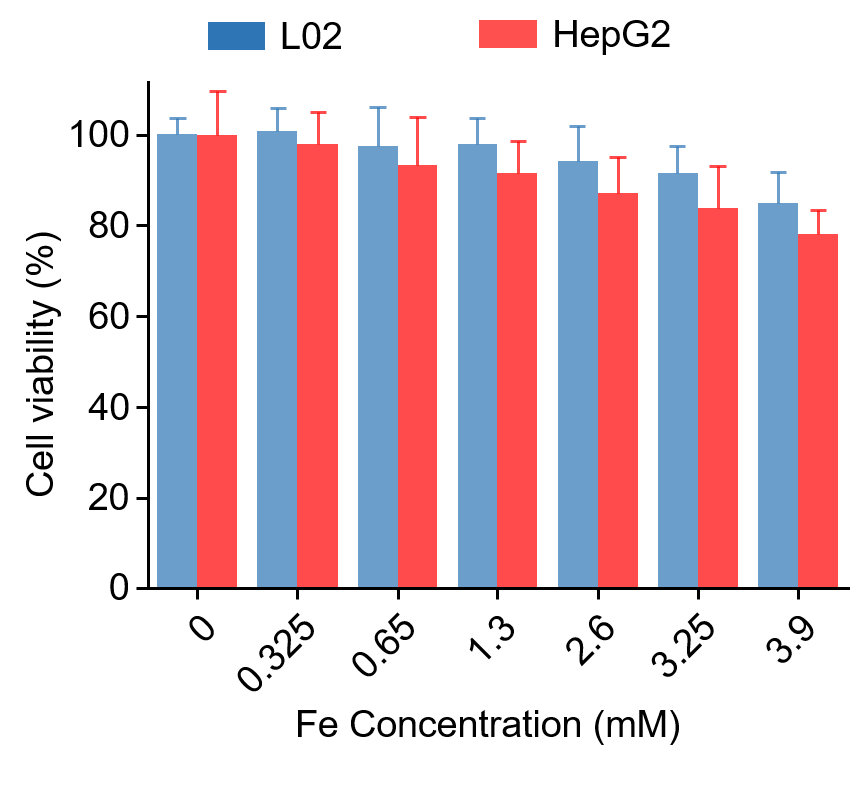


Figure S12. Cytotoxicity of FePt nanoprobes after the incubation with L02 and HepG2 cells for 24 h.


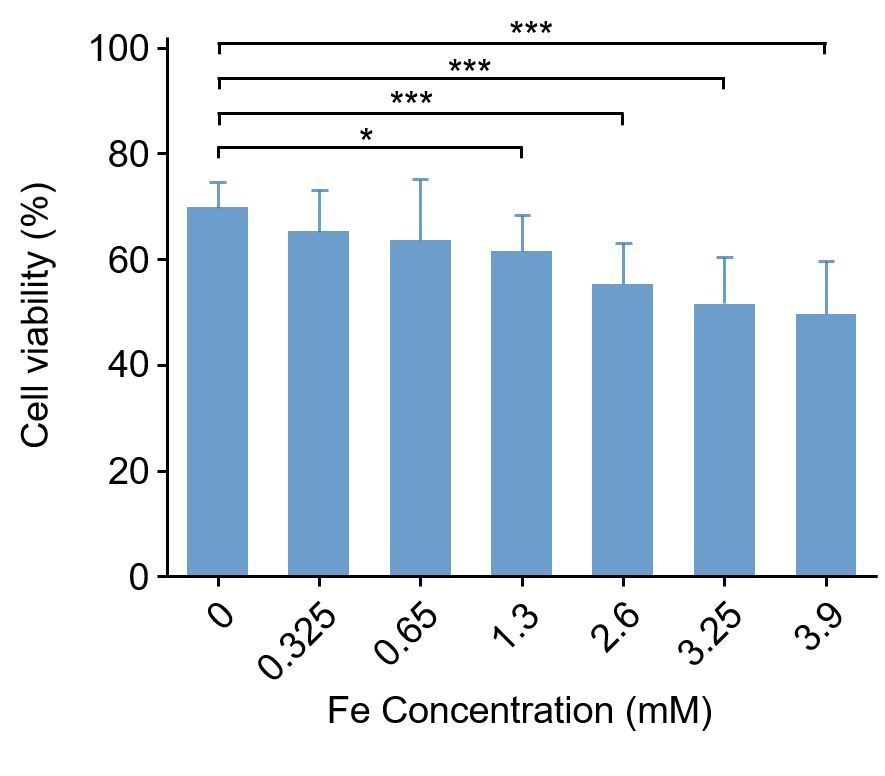


Figure S13. Cell viabilities of HepG2 cells after X-ray irradiation (4 Gy) with different concentrations of FePt nanoprobes (*, *P* < 0.05. ***, *P* < 0.001).


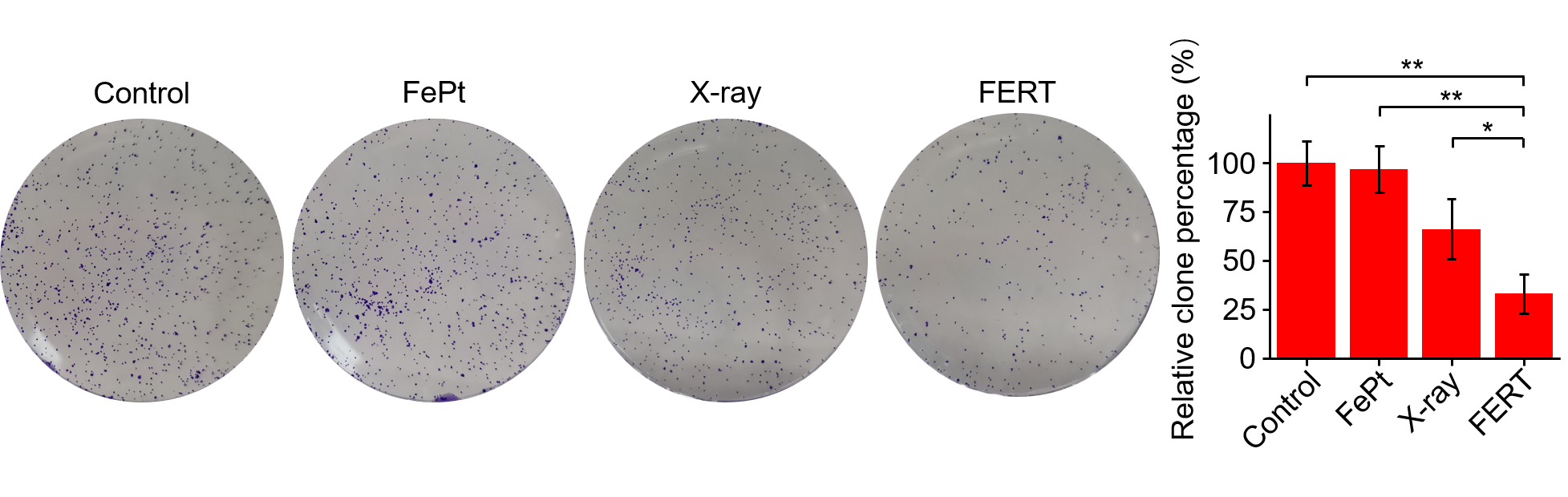


Figure S14. Colony formation assays after different treatments (**, *P* < 0.01. **, *P* < 0.01. *, *P* < 0.05).


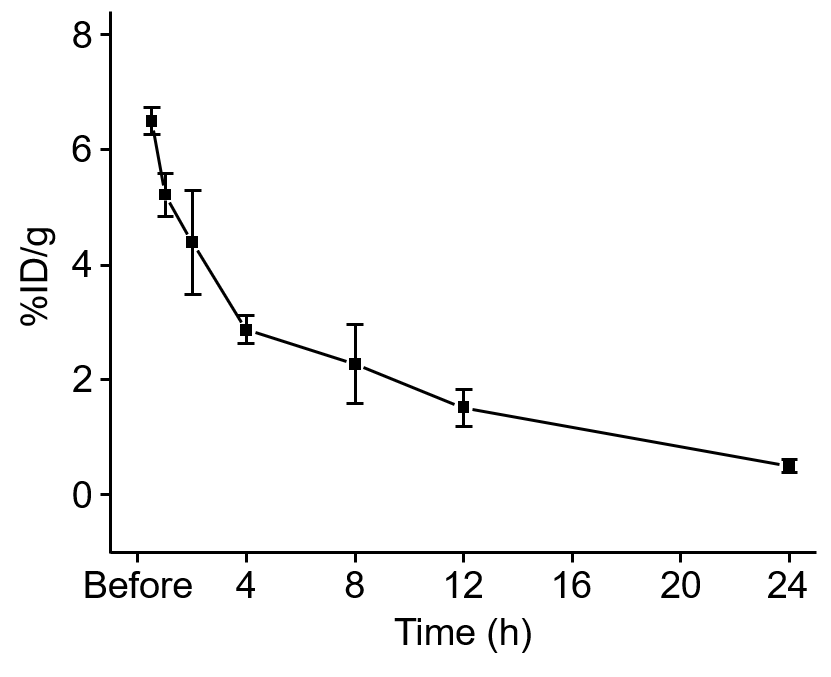


Figure S15. In vivo blood circulation by quantifying Pt concentration at different time points after intravenous injection of FePt nanoprobes.


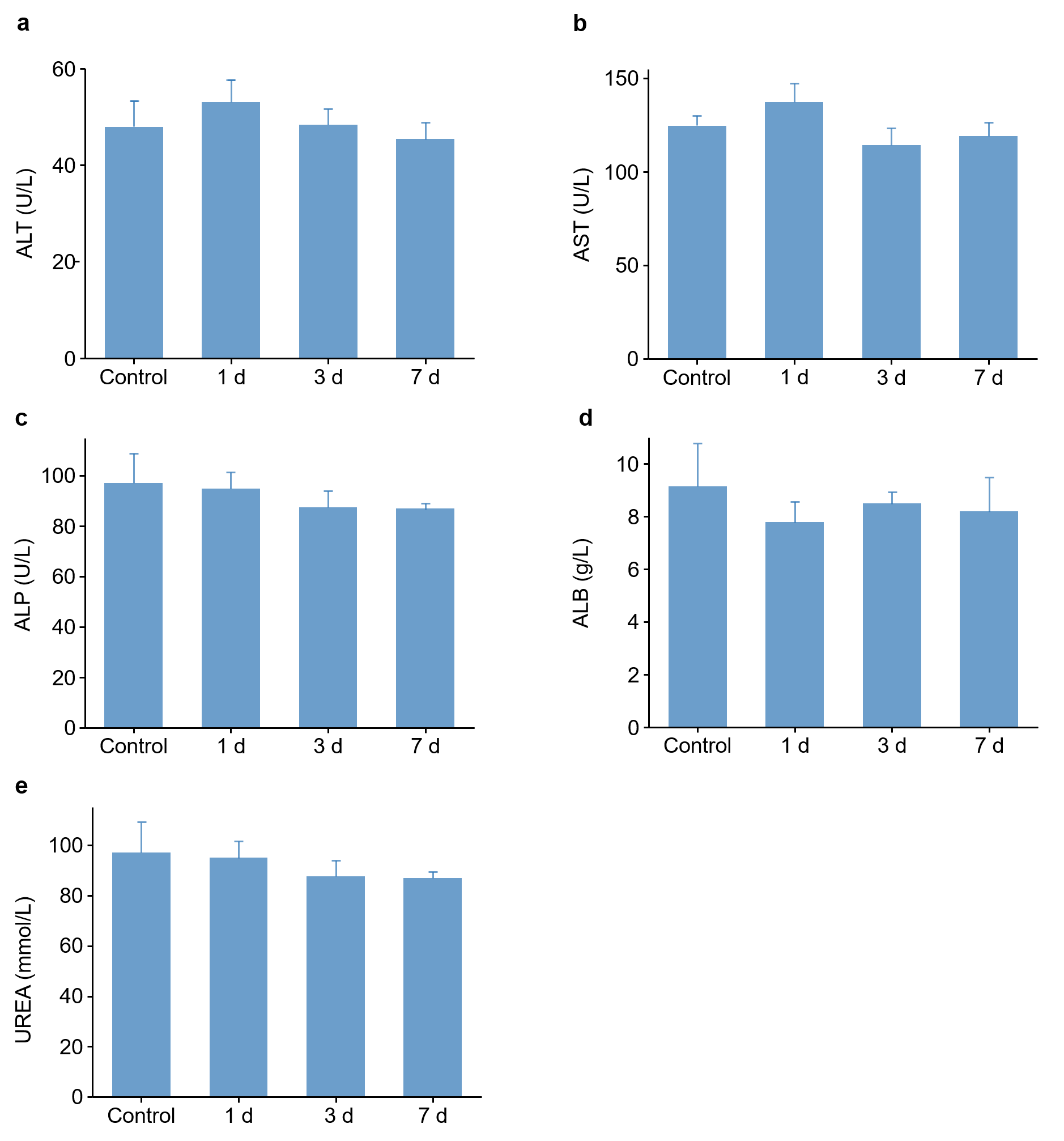


Figure S16. Serum biochemical indexes including ALT (a), AST (b), ALP (c), ALB (d) and urea (e) of the mice intravenously injected with FePt nanoprobes.


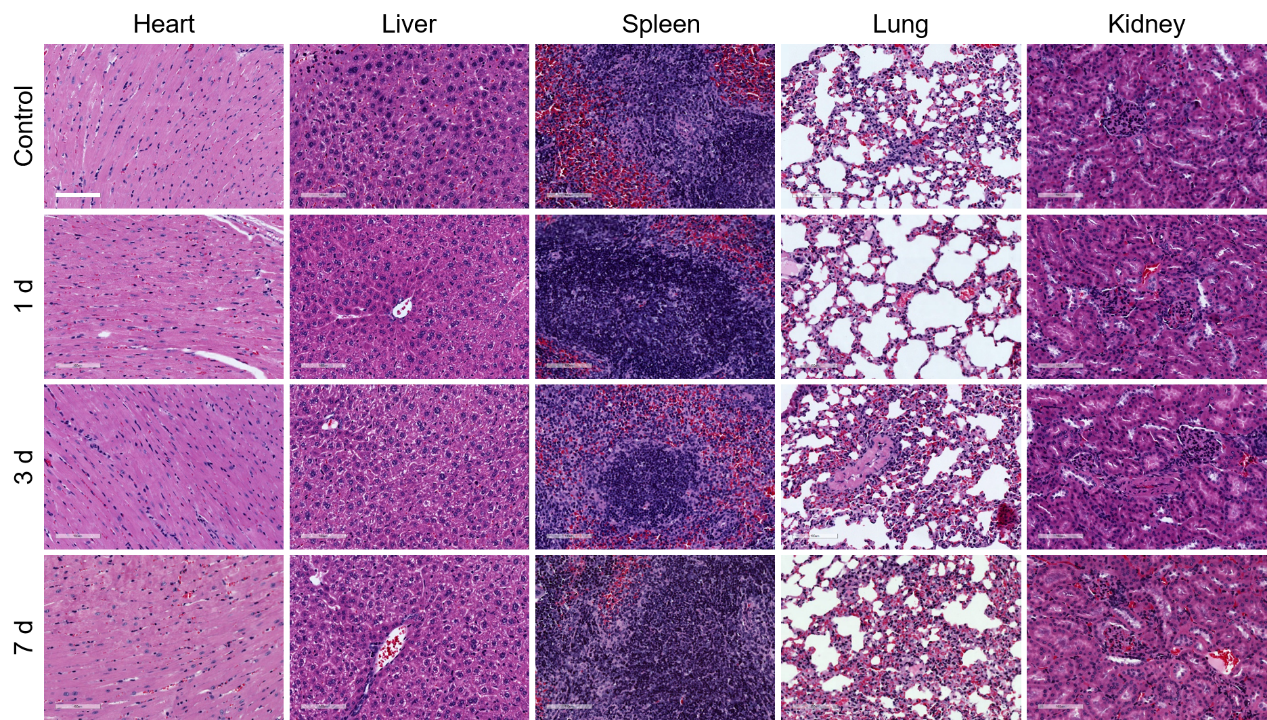


Figure S17. H&E staining images of main organs (heart, liver, spleen, lung and kidney) collected from mice after intravenous administration FePt nanoprobes (scale bar is 100 μm).


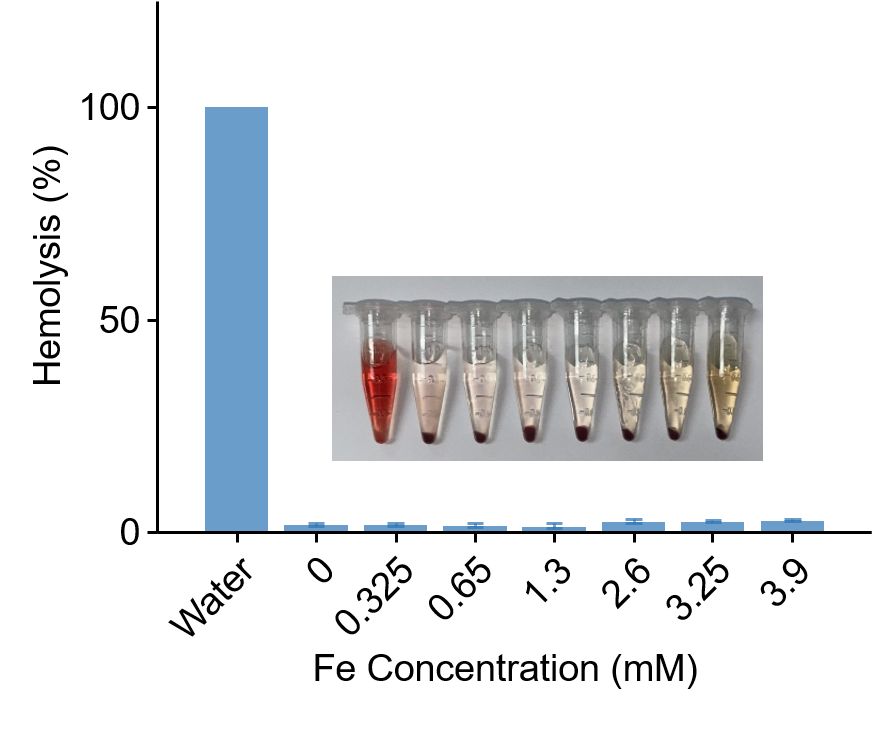


Figure S18. Hemolysis assays of FePt nanoprobes.


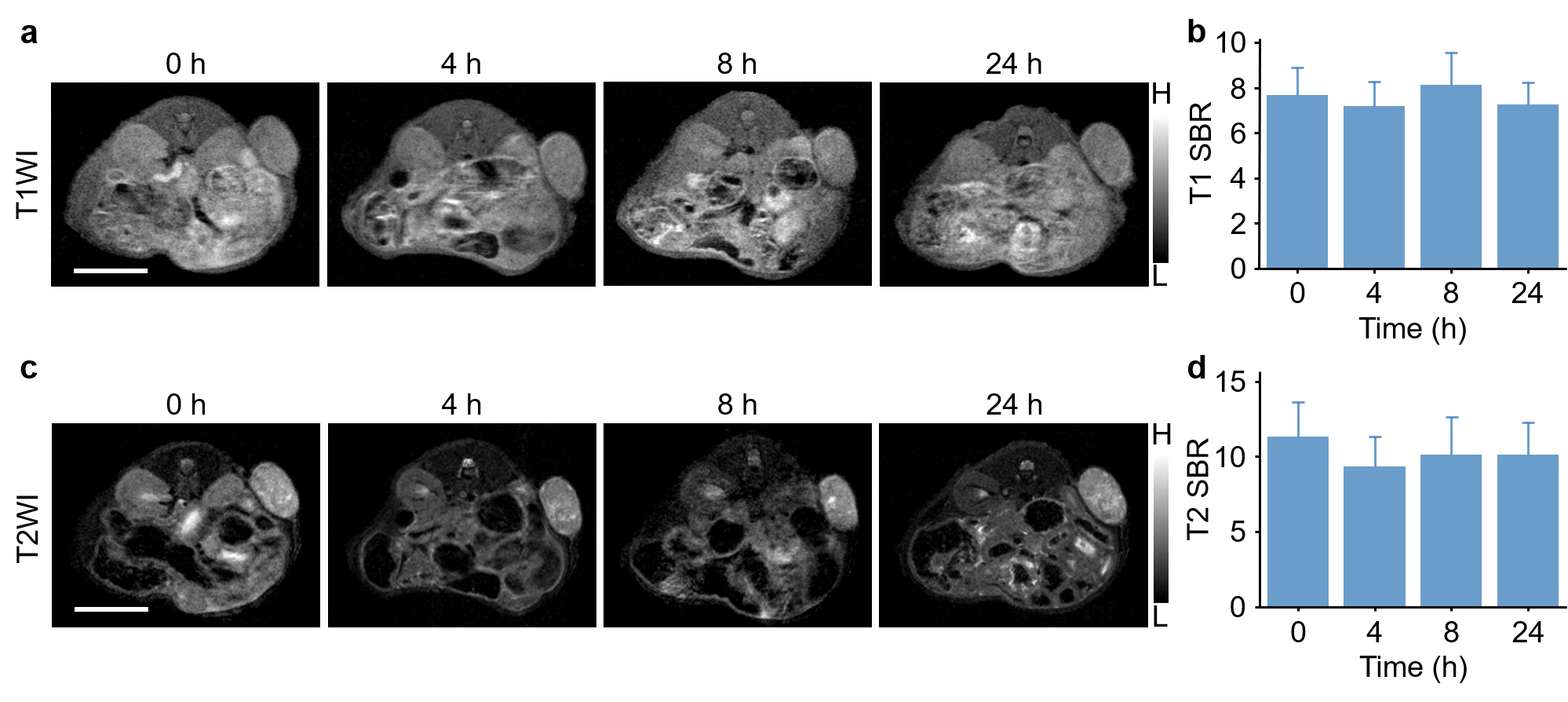


Figure S19. *In vivo* MRI of the tumors after intravenously injection of Fe_3_O_4_ nanoparticles. (a, b) *In vivo* T1WI of HepG2 tumors at various timepoints (scale bar: 1 cm) (a), and the corresponding T1 SBRs of the tumor area (b). (c, d) *In vivo* T2WI of HepG2 tumors at various timepoints (scale bar: 1 cm) (c), and the corresponding T2 SBRs of the tumor area (d).


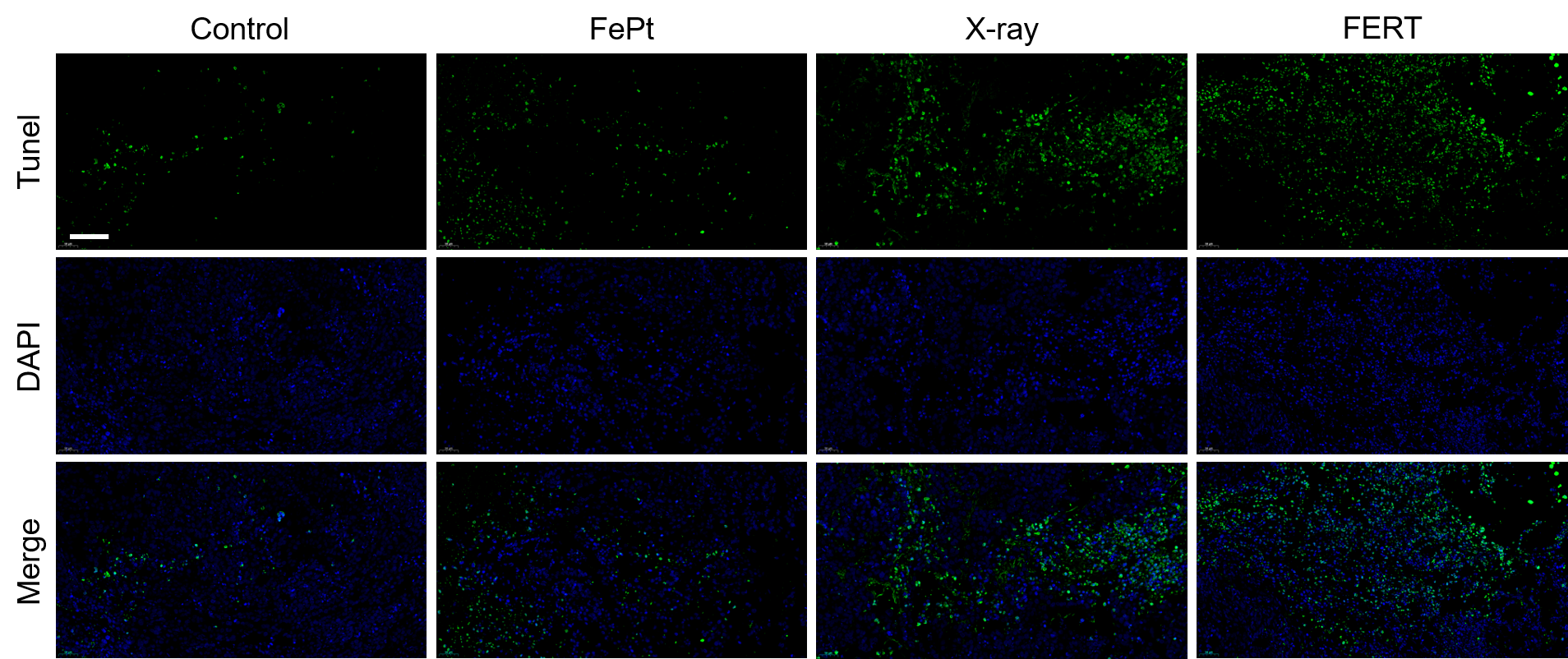


Figure S20. Tunel immunofluorescence of tumor slices collected from various groups of mice. （Scale bar: 100 μm）


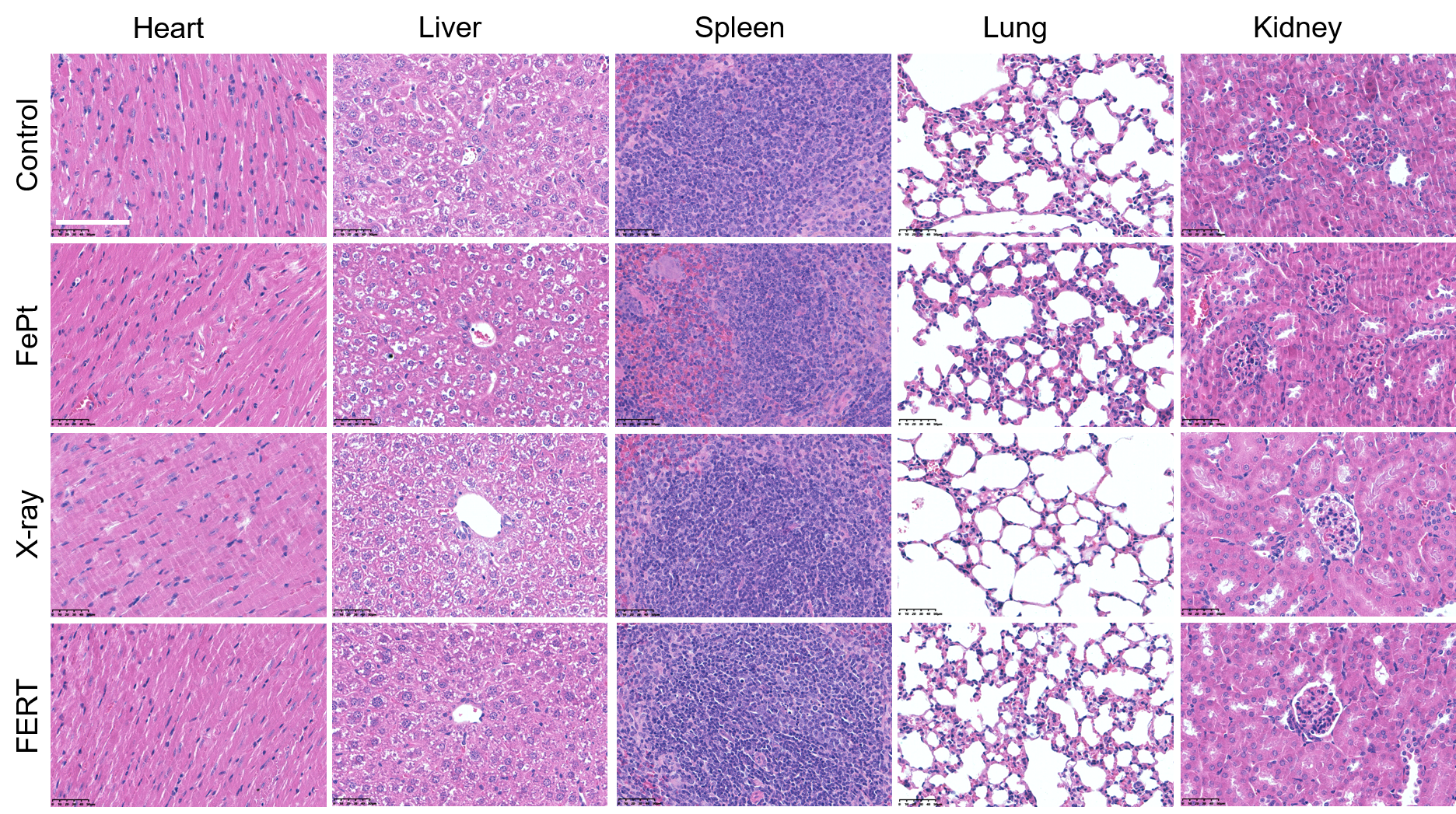


Figure S21. H&E staining images of main organs (heart, liver, spleen, lung and kidney) collected from mice after intravenous administration FePt nanoprobes (scale bar is 100 μm).
